# Influence of the surface viscous stress on the pinch-off of free surfaces loaded with nearly-inviscid surfactants

**DOI:** 10.1038/s41598-020-73007-1

**Published:** 2020-09-30

**Authors:** A. Ponce-Torres, M. Rubio, M. A. Herrada, J. Eggers, J. M. Montanero

**Affiliations:** 1 Depto. de Ingeniería Mecánica, Energética y de los Materiales and Instituto de Computación Científica Avanzada (ICCAEx), Universidad de Extremadura, 06006 Badajoz, Spain; 2 Depto. de Mecánica de Fluidos e Ingeniería Aeroespacial, Universidad de Sevilla, 41092 Sevilla, Spain; 3 School of Mathematics, University of Bristol, Fry Building, Bristol, BS8 1UG UK

**Keywords:** Fluid dynamics, Applied physics

## Abstract

We analyze the breakup of a pendant water droplet loaded with SDS. The free surface minimum radius measured in the experiments is compared with that obtained from a numerical solution of the Navier–Stokes equations for different values of the shear and dilatational surface viscosities. This comparison shows the small but measurable effect of the surface viscous stresses for sufficiently small spatiotemporal distances from the breakup point, and allows to establish upper bounds for the values of the shear and dilatational viscosities. We study numerically the distribution of Marangoni and viscous stresses over the free surface as a function of the time to the pinching, and describe how surface viscous stresses grow in the pinching region as the free surface approaches its breakup. When Marangoni and surface viscous stresses are taken into account, the surfactant is not swept away from the thread neck in the time interval analyzed. Surface viscous stresses eventually balance the driving capillary pressure in in the pinching region for small enough values of the time to pinching. Based on this result, we propose a scaling law to account for the effect of the surface viscosities on the last stage of temporal evolution of the neck radius.

## Introduction

Soluble surfactants play a fundamental role in many microfluidic applications^1^. For instance, it is well-known that surfactants can stabilize both foams and emulsions due to Marangoni convection effects^2–4^. The surface viscosity of surfactant monolayers is also believed to play a significant role in such stabilization. In fact, the drainage time during the coalescence of two bubbles/droplets can considerably increase due to the monolayer viscosity^5^. However, there are serious doubts about whether small-molecule surfactants commonly used in microfluidic applications exhibit measurable surface viscosities. For instance, Zell et al.^6^ reported that the surface shear viscosity of Sodium Dodecyl Sulfate (SDS) was below the sensitivity limit of their experimental technique ($\sim 10^{-8}$ Pa s m). This raises doubts about the role played by surface shear rheology in the stability of foams and emulsions treated with soluble surfactants.

The disparity among the reported values of shear and dilatational viscosities of both soluble and insoluble surfactants reflects the complexity of measuring such properties. The lack of precise information about these values, as well as the mathematical complexity of the calculation of the surface viscous stresses, has motivated that most of the experimental and theoretical works in microfluidics do not take into account those stresses. However, one can reasonably expect surface viscosity to considerably affect the dynamics of interfaces for sufficiently small spatiotemporal scales even for nearly-inviscid surfactants^7^. A paradigmatic example of this is the pinch-off of an interface covered with surfactant^7^, where both the surface-to-volume ratio and surface velocity can diverge for times and distances sufficiently close to this singularity.

In the pinching of a Newtonian liquid free surface, the system spontaneously approaches a finite-time singularity, which offers a unique opportunity to observe the behavior of fluids with arbitrarily small length and time scales. This property and its universal character (insensitivity to both initial and boundary conditions) turn this problem into an ideal candidate to question our knowledge of fundamental aspects of fluid dynamics. Both theoretical^8–12^ and experimental^7,13–15^ studies on the free surface pinch-off have traditionally considered the dependence of the free surface minimum radius, $R_{\text{min}}$, with respect to the time to the pinching, $\tau$, as an indicator of the relevant forces arising next to the pinching spatiotemporal coordinate. For small viscous effects, the thinning of the liquid thread passes through an inertio-capillary regime characterized by the power law

1 $$\begin{aligned} R_{\text{min }}=A \left( \frac{\sigma }{\rho }\right) ^{1/3} \tau ^{2/3}, \end{aligned}$$

where $\sigma$ and $\rho$ are the liquid surface tension and density, respectively^9,16^. The dimensionless prefactor *A* can exhibit a complex, nonmonotonic behavior over many orders of magnitude in $\tau$. In fact, its asymptotic value $A\simeq 0.717$ is never reached because there are very long-lived transients, and then viscous effects take over^17^.

The addition of surfactant confers a certain degree of complexity on Newtonian liquids, which may lead to unexpected behaviors during the pinch-off of their free surfaces. For instance, Marangoni stress can produce microthread cascades during the breakup of interfaces loaded with surfactants^18^. It is still a subject of debate whether surfactants are convected away from the pinching region. In that case, the system would follow the self-similar dynamics of clean interfaces at times sufficiently close to the breakup^7,13,19–25^. The persistence of a surfactant monolayer in the pinching of an interface potentially entails the appearance of several effects. The first and probably more obvious is the so-called solutocapillarity, i.e., the local reduction of the surface tension due to the presence of surface-active molecules^24,26^. The other effect that has been accounted for is the Marangoni stress induced by the surface tension gradient due to uneven distribution of surfactant along the free surface^12,18–20,22,27–32^. However, some other effects might be considered in the vicinity of the pinching region as well. Among them, the shear and dilatational surface viscosities have already been shown to affect considerably the breakup of pendant drops covered with insoluble (viscous) surfactants^7^.

SDS is one of the most commonly used surfactants in microfluidic experiments. The adsorption/desorption times of SDS are several orders of magnitude larger than the characteristic time of the breakup of free surfaces enclosing low-viscosity liquids. This allows one to regard SDS as an insoluble surfactant, which considerably simplifies the problem. Under the insolubility condition, bulk diffusion and adsorption/desorption processes can be ruled out. Due to its small molecular size, the SDS monolayer is assumed to exhibit a Newtonian behavior^33^. In addition, the sphere-to-rod transition of SDS micelles (and its associated viscoelastic behavior) does not take place unless some specific salt is added to the solution^34^. Therefore, viscoelastic effects are not expected to come up even for concentrations larger than the cmc.

Surface viscosities of small-size surfactant molecules, such as SDS, are believed not to affect the breakage of a pendant drop due to their small values. However, and as mentioned above, the surface-to-volume ratio diverges in the vicinity of the pinching region and, therefore, surface viscous effects can eventually dominate both inertia and viscous dissipation in the bulk of that region. In addition, the surface tension is bounded between the values corresponding to the clean free surface and the maximum packaging limit, while surface velocity can diverge at the pinch-off singularity. This suggests that surface viscous stresses (which are proportional to the surface velocity gradient) can become comparable with, or even greater than, Marangoni stress (which is proportional to surface tension gradient) in the pinching region for times sufficiently close to the breakup. One can hypothesize that surface viscous stresses can eventually have a measurable influence on the evolution of the free surface even for very low-viscosity surfactants. This work aims to test this hypothesis. The comparison between numerical simulations and experimental data will allow us to determine upper bounds for both the shear and dilatational viscosities of SDS. We will propose a scaling law which reflects the balance between the driving capillary force and the resistant surface viscous stresses in the last stage of the free surface breakup.

## Results and discussion

In this work, experiments were conducted with unprecedented spatiotemporal resolution to determine the free surface minimum radius as a fuction of the time to the pinching. The experimental results were compared with a numerical solution of the full Navier–Stokes equations which includes the effects of the shear and dilatational viscosities. The experimental procedure, theoretical model, and numerical method are described in “Methods” section.

**Figure 1:**
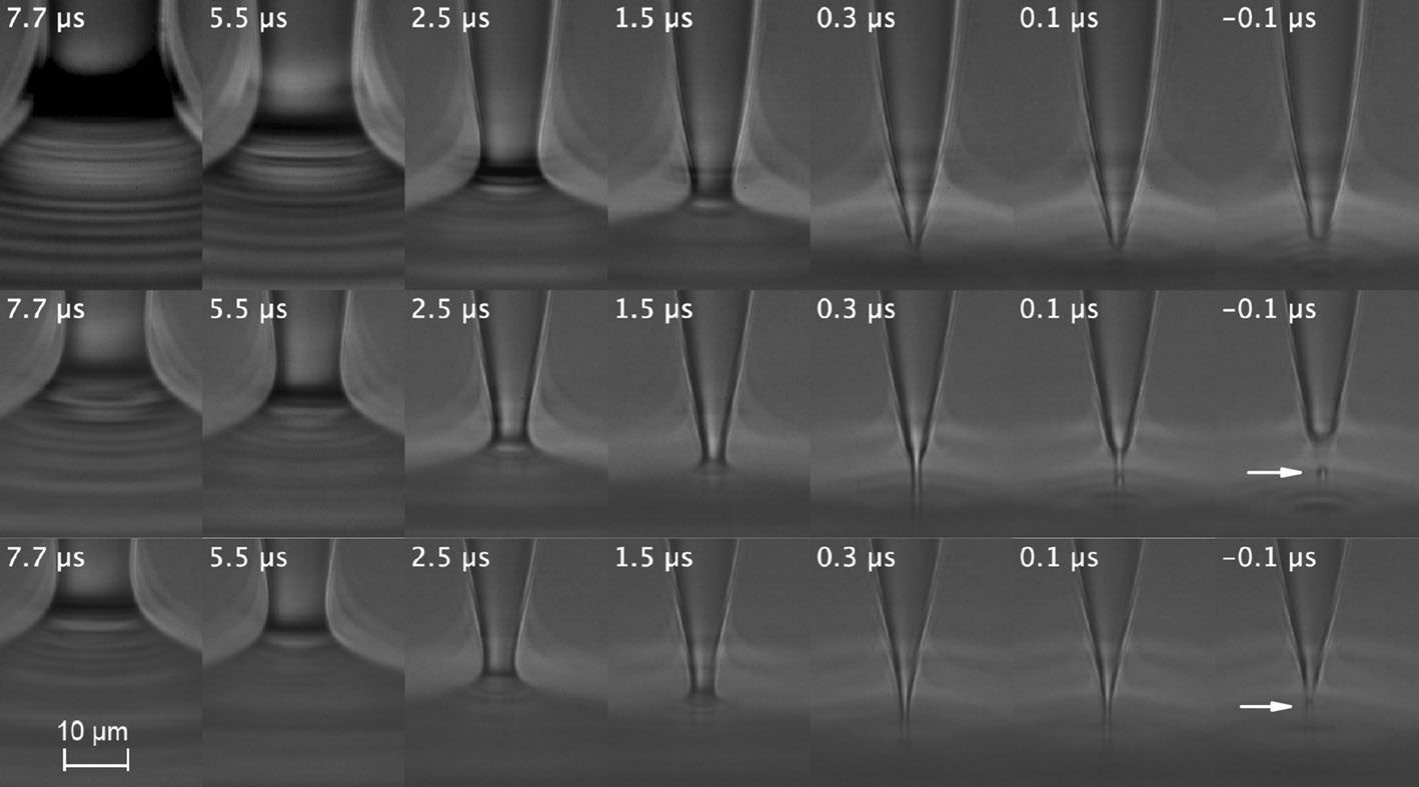
(From top to bottom) Pinch-off of a drop of DIW, DIW+SDS 0.8cmc, and DIW+SDS 2cmc. The labels indicate the time to the pinching with an error of ±100 ns. The arrows point to the subsatellite droplets.

Figure 1 shows images of the pinch-off of a drop of deionized water (DIW), DIW+SDS 0.8cmc, and DIW+SDS 2cmc. A microthread forms next to the pinching point when the surfactant is added. The breakup of that microthread produces a tiny subsatellite droplet 1–2 $\upmu$m in diameter. This droplet is significantly smaller than that observed in previous experiments with 5-cSt silicone oil in the absence of surfactant, which seems to confirm that the silicone oil subsatellite droplet was formed by viscoelastic effects^35^.

**Figure 2:**
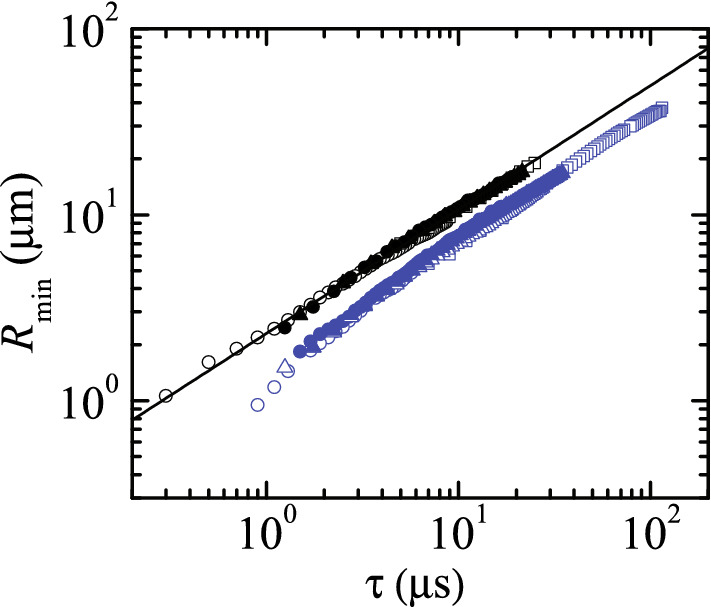
$R_{\text{min }}(\tau )$ for the breakup of a pendant drop of DIW and DIW+SDS 0.8cmc. The black and blue symbols are the experimental data for DIW and DIW+SDS 0.8cmc, respectively. The different symbols correspond to experiments visualized with different magnifications and recording speeds. The open and solid symbols correspond to experiments conducted with a cylindrical feeding capillary $R_0=115$ and 205 $\upmu$m in radius, respectively. The solid line is the power law (1) with $A\simeq 0.55$.

Figure 2 shows the free surface minimum radius, $R_{\text{min }}$, as a function of the time to the pinching, $\tau$, for experiments conducted with two feeding capillary radii $R_0$ (see“Methods” section). The agreement among the results obtained for the same liquid shows both the high reproducibility of the experiments and the universal character (independency from $R_0$) of $R_{\text{min }}(\tau )$ for the analyzed time interval. In fact, the differences between the results obtained with $R_0=115$ and 205 $\upmu$m are smaller than the effect attributed to the surface viscosities, as will be described below. The results for DIW follow the scaling law (1) with $A\simeq 0.55$.

**Figure 3:**
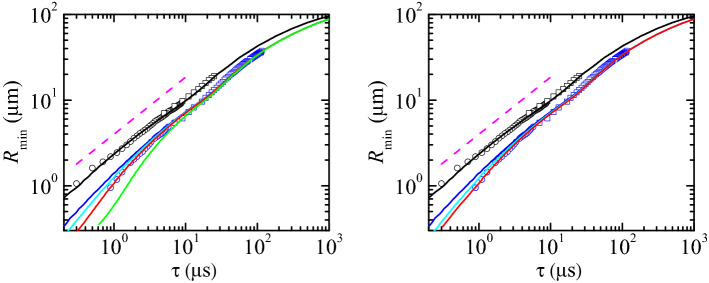
$R_{\text{min }}(\tau )$ for the breakup of a pendant drop of DIW and DIW+SDS 0.8cmc. The black and blue symbols are the experimental data for DIW and DIW+SDS 0.8cmc, respectively. The different symbols correspond to experiments visualized with different magnifications. The black solid line and magenta dashed line correspond to the simulation and the power law $R_{\text{min }}(\tau )\sim \tau ^{2/3}$ for DIW, respectively. (Left) The colored solid lines correspond to simulations of DIW+SDS 0.8cmc for $\mu _2^{S*}=0$ and $\mu _1^{S*}=0$ (blue), $5 \times 10^{-10}$ (cyan), $1.2 \times 10^{-9}$ (red), and $5 \times 10^{-9}$ Pa s m (green). (Right) The colored solid lines correspond to simulations of DIW+SDS 0.8cmc for $\mu _1^{S*}=0$ and $\mu _2^{S*}=0$ (blue), $10^{-7}$ (cyan), $6 \times 10^{-7}$ Pa s m (red). All the numerical results were calculated for $B=3.396 \times 10^{-3}$, $\text {Oh}=0.01510$, ${\widehat{\Gamma }}_{\text{cmc }}=1.002$, and Pe$^S=7.730 \times 10^{4}$ (see “Methods” section). In the left-hand graph, the colored solid lines correspond to Oh$_2^{S*}=0$ and Oh$_1^{S*}=0$ (blue), $6.563 \times 10^{-5}$ (cyan), $1.575 \times 10^{-4}$ (red), and $6.563 \times 10^{-4}$ (green) (see “Methods” section). In the right-hand graph, the colored solid lines correspond to Oh$_1^{S*}=0$ and Oh$_2^{S*}=0$ (blue), $1.313 \times 10^{-2}$ (cyan), $7.876 \times 10^{-2}$ (red).

As can be seen in Fig.  3, there is a remarkable agreement between the experiments and numerical simulations for the pure DIW case for times to the pinching as small as $\sim 300$ ns, which constitutes a stringent validation of both experiments and simulations. When SDS is dissolved in water, it creates a monolayer which substantially alters the pinch-off dynamics. The function $R_{\text{min }}(\tau )$ takes smaller values than in the pure DIW case over the entire process due to the reduction of the surface tension. More interestingly, if only solutocapillarity and Marangoni convection are considered in the numerical simulations (blue solid lines), there is a measurable deviation with respect to the experimental results for $R_{\text{min }}(\tau )\lesssim 5$ $\upmu$m. Specifically, the free surface in the experiment evolves towards its pinching slower than in the numerical simulation. We added surface viscous stresses (see “Methods” section) to the simulation to reproduce the entire range of experimental data. To this end, we set to zero one of the surface viscosities and modulated the other. In this way, one can establish upper bounds of both the surface shear $\mu _1^{S*}$ and dilatational $\mu _2^{S*}$ viscosity at the cmc (see “Methods” section).

The numerical results fit the experimental measurements for $\mu _1^{S*}=1.2 \times 10^{-9}$ Pa s m and $\mu _2^{S*}=0$ (Fig. 3-left) or $\mu _1^{S*}=0$ and $\mu _2^{S*}=6\times 10^{-7}$ Pa s m (Fig. 3-right). As can be observed, the optimum value of the dilatational viscosity $\mu _2^{S*}$ is more than two orders of magnitude larger than that of the shear viscosity $\mu _1^{S*}$. This means that the effect of the dilatational viscosity is much smaller than that of the shear viscosity. If one assumes that the values of both viscosities are commensurate with each other, the dilatational viscosity plays a negligible role in the filament thinning. This result has practical consequences because it means that the breakup of a pendant drop can be used to measure the shear surface viscosity of a nearly-inviscid surfactant monolayer. The value $\mu _1^{S*}=1.2 \times 10^{-9}$ Pa s m is consistent with the results obtained by Zell et al.^6^, who concluded that the shear viscosity of SDS in DIW must take values below $10^{-8}$ Pa s m (the sensitivity limit of their technique).

**Figure 4:**
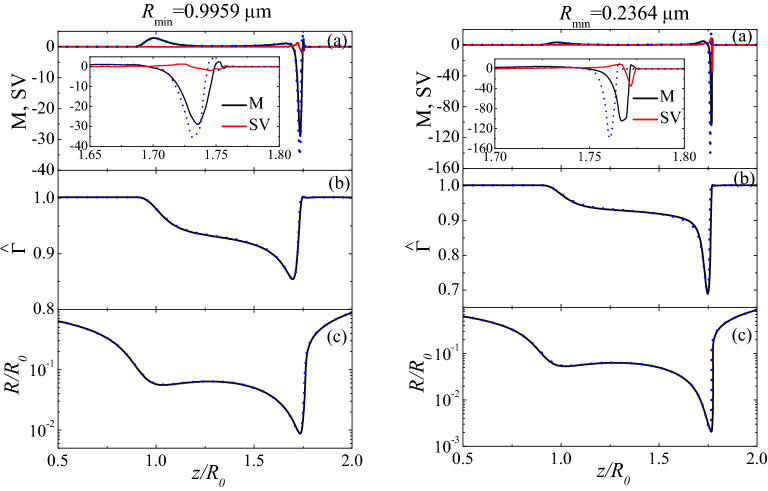
Axial distribution of the Marangoni stress M and tangential shear viscous stress SV (**a**), surfactant surface concentration ${\widehat{\Gamma }}$ (**b**), and free surface radius $R/R_0$ (c) for DIW+SDS 0.8cmc. The solid lines are the results for $\{\mu _1^{S*}=1.2\times 10^{-9}$, $\mu _2^{S*}=0$ Pa s m$\}$, while the dotted lines correspond to the the Marangoni stress M for $\mu _1^{S*}=\mu _2^{S*}=0$ (in the left-hand graphs, $R_{\text{min }}=0.9836$ $\upmu$m for $\mu _1^{S*}=\mu _2^{S*}=0$; in the right-hand graphs, $R_{\text{min }}=0.2268$ $\upmu$m for $\mu _1^{S*}=\mu _2^{S*}=0$). The results were calculated for $B=3.396 \times 10^{-3}$, Oh=0.0151, ${\widehat{\Gamma }}_{\text{cmc }}=1.002$, Pe$^S=7.73 \times 10^{4}$, Oh$_2^{S*}=0$, and Oh$_1^{S*}=1.575 \times 10^{-4}$ (solid lines) and 0 (dotted lines) (see “Methods” section).

Figure 4 shows the values of the axial distribution of the Marangoni stress M and tangential shear viscous stress SV, the surfactant surface concentration ${\widehat{\Gamma }},$ and the free surface radius $R/R_0$ for DIW+SDS 0.8cmc. Here,

2 $$\begin{aligned} \text {M}\equiv \mathbf{t}\cdot \varvec{\nabla }^S{\hat{\sigma }},\quad \text {SV}\equiv \mathbf{t}\cdot \{\varvec{\nabla }^S[-\text {Oh}_1^S(\varvec{\nabla }^S\cdot \mathbf{v})]+2\varvec{\nabla }^S\cdot (\text {Oh}_1^S{{\mathbf {\mathsf{{D}}}}}^S)\}, \end{aligned}$$

where $\text {Oh}_{1}^S$ is the superficial Ohnesorge number defined in terms of the surface shear viscosity (see “Methods” section). The relative importance of the shear viscosity increases as the minimum radius decreases. The presence of shear viscosity slightly reduces the magnitude of the Marangoni stress. The viscous surface stress hardly alters the surfactant distribution and the free surface shape.

As mentioned in the Introduction, there is still a certain controversy about whether surfactants are convected away from the pinching region^7,13,19–25^. Our results show that, when Marangoni and surface viscous stresses are taken into account, the surfactant is not swept away from the thread neck in the time interval analyzed (${\widehat{\Gamma }}\gtrsim {0.7}$ in this region). These stresses operate in a different way but collaborate to keep the surfactant in the vicinity of the pinching point. Marangoni stress tries to restore the initial uniform surfactant concentration, while surface viscosity opposes to the variation of the surface velocity, and, therefore, to the extensional flow responsible for the surfactant depletion that would occur in the absence of Marangoni and viscous stresses.

Interestingly, the free surface shape for $\mu _1^{S*}=\mu _2^{S*}=0$ is practically the same as that with the adjusted value of $\mu _2^{S*}$. This indicates that surface viscosity simply delays the time evolution of that shape. In fact, the values of the minimum radius obtained with and without surface viscosity significantly differ from each other when they are calculated at the same time to the pinching. For instance, $R_{\text{min }}=0.24$ and 0.42 $\upmu$m at $\tau \simeq 0.25$ $\upmu$s for $\{\mu _1^{S*}=1.2 \times 10^{-9}$ Pa s m, $\mu _2^{S*}=0\}$ and $\mu _1^{S*}=\mu _2^{S*}=0$, respectively. However, the free surface shapes are practically the same if they are compared when the same value $R_{\text{min }}=0.24$ $\upmu$m of the minimum radius is reached. In addition, the surfactant density distribution is not considerably affected by the surface viscosity. We can conclude that the surface viscosities of the SDS monolayer hardly alter the satellite droplet diameter and the amount of surfactant trapped in it. In this sense, solutocapillarity and Marangoni convection are the major factors associated with the surfactant^12^. These results differ from those obtained for a much more viscous surfactant^7^.

We now study how the scaling of the minimum radius depends on the surfactant viscosities. In general, we have $R_{\text{min }}=f(\tau ,\mu _{1,2}^S)$. Assume that we can write this equation in the form $R_{\text{min }}=R_s H(\tau /\tau _s)$, where $R_s$ and $\tau _s$ are the length and time scales associated with the surface viscosities, respectively. We suppose that these scales depend on the viscosities as

3 $$\begin{aligned} R_s=A (\mu _{1,2}^{S*})^{\alpha }, \quad \tau _s=B (\mu _{1,2}^{S*})^{\beta }. \end{aligned}$$

The cross-over function $H(\xi )$ behaves as $H(\xi )\sim \xi ^{2/3}$ for $\xi \gg 1$ (inviscid limit) and $H(\xi )\sim \xi ^{\gamma }$ for $\xi \ll 1$ (viscous regime), with a crossover at $\xi \sim 1$. Therefore, $R_{\text{min }}=A B^{-2/3} (\mu _{1,2}^S)^{\alpha -2\beta /3}\tau ^{2/3}$ in the inviscid limit. Assuming that $R_{\text{min }}\sim \tau ^{2/3}$ in that limit, we conclude that $\alpha =2\beta /3$.

The value of the exponent $\beta$ can be guessed from the balance of forces. Both Marangoni and surface viscous stresses delay the free surface pinch-off (Fig. 3) acting against the driving capillary force. For sufficiently small values of $R_{\text{min }}$, the effect of surface viscous stresses become comparable to that caused by Marangoni stress (Fig. 4). The value of $R_{\text{min }}$ below which this occurs decreases as the surface viscosities decrease. Therefore, we expect surface viscous stresses to be commensurate with the driving capillary pressure in the pinch-off region for $R_{\text{min }}\rightarrow 0$.

**Figure 5:**
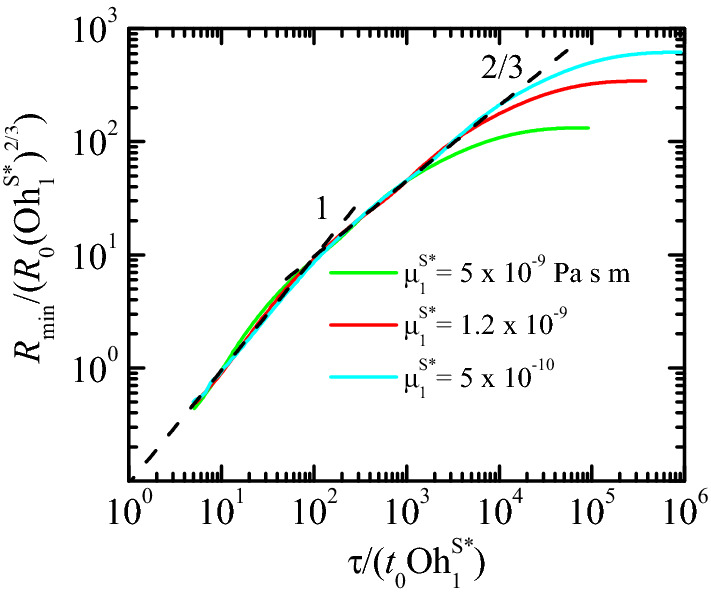
Dimensionless minimum radius $R_{\text{min }}/R_0$ as a function of the dimensionless time to the breakup, $\tau /t_0$, for the breakup of a pendant drop of DIW+SDS 0.8cmc. The labels indicate the values of the shear surface viscosity in each case. The dilatational surface viscosity was set to zero. The results were calculated for $B=3.396 \times 10^{-3}$, Oh = 0.0151, ${\widehat{\Gamma }}_{\text{cmc }}=1.0016$, and Pe$^S=7.73 \times 10^{4}$ (see “Methods” section).

The balance between the capillary pressure and the normal surface viscous stresses in Eq.  (10) yields $\sigma _0/R_s\sim \mu _{1,2}^{S*}/(R_s\tau _s)$, where we have taken into account that the variation of surface velocity scales as $(R_s/\tau _s)/R_s$ due to the continuity equation. The above balance allows us to conclude that $\beta =1$, and therefore $\alpha =2/3$. According to our analysis,

4 $$\begin{aligned} \frac{R_{\text{min }}}{(\mu _{1,2}^{S*})^{2/3}}\sim \left( \frac{\tau }{\mu _{1,2}^{S*}}\right) ^{\gamma }, \end{aligned}$$

in the viscous regime. According to our previous results (Fig.  3), we can assume that the dilatational viscosity plays a negligible role. Then, we have

5 $$\begin{aligned} \frac{R_{\text{min }}}{(\mu _{1}^{S*})^{2/3}}\sim \left( \frac{\tau }{\mu _{1}^{S*}}\right) ^{\gamma }, \end{aligned}$$

in surface viscosity-dominated regime. Figure 5 shows the results scaled with those exponents. The simulations show the transition from the inertio-capillary regime $R_{\text{min }}\sim \tau ^{2/3}$ to the asymptotic behavior given by power law $\gamma =1$. The asymptotic behavior $R_{\text{min }}\sim \tau$ coincides with that recently derived by Wee et al.^36^.

**Figure 6:**
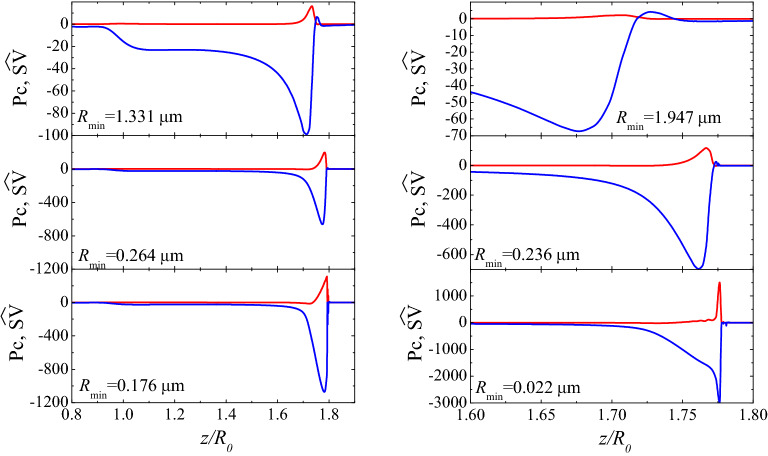
Axial distribution of the capillary pressure Pc (blue lines) and normal shear viscous stress $\widehat{\text {SV}}$ (red lines) for DIW+SDS 0.8cmc and three instants as indicated by the value of $R_{\text{min }}$. The left-hand and right-hand graphs correspond to $\{\mu _1^{S*}=5\times 10^{-9}$, $\mu _2^{S*}=0$ Pa s m$\}$ and $\{\mu _1^{S*}=1.2\times 10^{-9}$, $\mu _2^{S*}=0$ Pa s m$\}$, respectively. The results were calculated for $B=3.396 \times 10^{-3}$, Oh = 0.0151, ${\widehat{\Gamma }}_{\text{cmc }}=1.002$, Pe$^S=7.730 \times 10^{4}$, Oh$_2^{S*}=0$, and Oh$_1^{S*}=6.563 \times 10^{-4}$ (left-hand graphs) and $1.575 \times 10^{-4}$ (right-hand graphs) (see “Methods” section).

Figure 6 shows the axial distribution of the capillary pressure Pc and normal shear viscous stress $\widehat{\text {SV}}$ for DIW+SDS 0.8cmc at three instants as indicated by the value of $R_{\text{min }}$. Here,

6 $$\begin{aligned} \text {Pc}=-(\varvec{\nabla }^S\cdot \mathbf{n}){\hat{\sigma }}, \quad \widehat{\text {SV}}=\text {Oh}_1^S(\varvec{\nabla }^S\cdot \mathbf{n})(\varvec{\nabla }^S\cdot \mathbf{v}). \end{aligned}$$

We consider the shear viscous stress $\widehat{\text {SV}}$ because the results indicate that shear viscosity plays a more significant role than the dilatational one. The normal shear viscous stress becomes comparable with the capillary pressure as $R_{\text{min }}\rightarrow 0$.

To summarize, we studied both numerically and experimentally the breakup of a pendant water droplet loaded with SDS. We measured a delay of the droplet breakup with respect to that predicted when only solutocapillarity and Marangoni stress are accounted for. This delay is attributed to the role played by surface viscosities. When Marangoni and surface viscous stresses are accounted for, then surface convection does not sweep away the surfactant from the thread neck, at least in the time interval analyzed. The results show that surface viscous stresses have little influence on both the free surface position and the surfactant distribution along the free surface. Therefore, the size of the satellite droplet and the amount of surfactant accumulated in it are hardly affected by the surface viscosities. These results differ from those obtained for a much more viscous surfactant^7^. As the free surface approaches its breakup, an inertio-capillary regime gives rise to that in which surface viscous stresses become commensurate with the driving capillary pressure. We have proposed a scaling law to account for the effect of surface viscosities on $R_{\text{min }}(\tau )$ in this last regime.

In the presence of surfactant, both the simulations and experiments show the formation of a quasi-cylindrical filament near the pinching point for $\tau \lesssim 0.1$ $\upmu$s (see Figs. 1, 4c). This filament is the precursor of the subsatellite droplet formed later on in the experiments. For $\tau \lesssim 0.1$ $\upmu$s, a bead seems to protrude from the filament in the experiments, which gives rise to the formation of the subsatellite droplet. The temporal resolution of the image acquisition system does not enable describing this process to determine the instant at which the filament bulges. In the simulations, we did not observe the filament protrusion preceding the formation of the subsatellite droplet. Therefore, discrepancies between the simulations and experiments associated with the growth of subsatellite droplets can arise for $\tau \lesssim 0.1$ $\upmu$s. The surface viscosities are estimated by fitting the numerical solution to the experiments for $\tau \gtrsim 1$ $\upmu$s. Therefore, this fitting is not expected to be affected by those discrepancies. However, Figs. 4, 5 and 6 show numerical results for times to the pinching down to 0.1–0.2 $\upmu$s. There can be differences between the experiments and simulations for those times. These differences can be attributed not only to the spatial resolution of the numerical method, but also to possible physical effects not accounted for in the governing equations and brought to light by the extremely small spatial and temporal scales, such as surface-active impurities in the free surface^37^, non-linear contributions to the dependency of the surface viscosities on the surfactant concentration, and interfacial rheology.

The pinching of an interface is a singular phenomenon that allows us to test theoretical models under extreme conditions. The vanishing spatiotemporal scales reached by the system as the interface approaches its breakup unveil physical effects hidden in phenomena occurring on much larger scales. This work is an example of this. Surface viscous stresses become relevant in the vicinity of the pinching region long before thermal fluctuations become significant^38,39^, even for practically inviscid surfactants, such as SDS. Besides, the effect of the dilatational surface viscosity on the thinning has shown to be negligible with respect to the shear viscosity. In this sense, the surfactant-laden pendant droplet can be seen as a very sensitive surfactometer to determine the values of the surface shear viscosity, which constitutes a difficult problem^40^. A series of experiments for different surfactant concentrations and needle radii may lead to accurate measurements of $\mu _1^{S}(\Gamma )$ characterizing the behavior of low-viscosity surfactants.

## Methods

### Theoretical model

**Figure 7:**
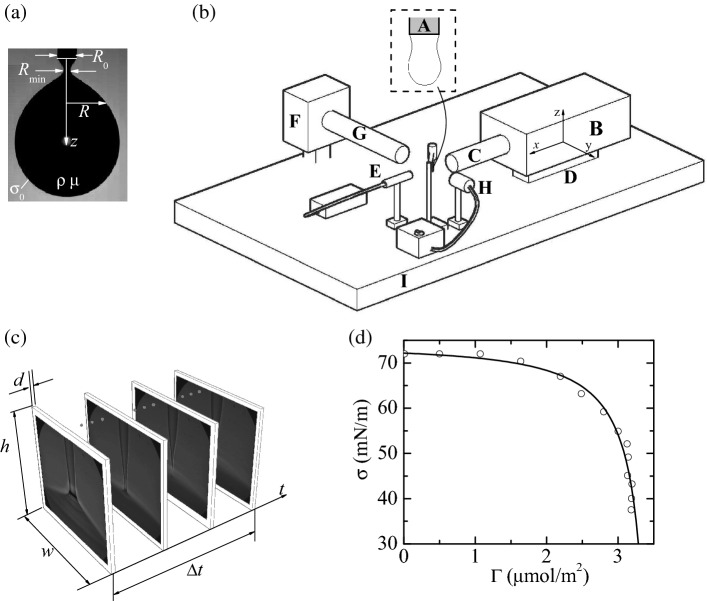
(**a**) Image of a pendant drop in the experiments right before its breakup. (**b**) Experimental setup: feeding capillary (A), ultra-high speed video camera (B), optical lenses (C), triaxial translation stage (D), laser (E), optical trigger (F), optical lenses (G), white backlight (H), and anti-vibration isolation system (I). (**c**) Spatio-temporal hypervolume analyzed in the experiment: image width $w=94$ $\upmu$m, height $h=78$ $\upmu$m, depth of field $d=0.48$ $\upmu$m and time $\Delta t=36$ $\upmu$s elapsed during the experiment. (**d**) Experimental values of the surface tension $\sigma$ versus the surface surfactant concentration $\Gamma$ for SDS in DIW (symbols)^45^. The line corresponds to the fit (13) to those values.

Consider a liquid drop of density $\rho$ and viscosity $\mu$ hanging on a vertical capillary (needle) of radius $R_0$ due to the action of the (equilibrium) surface tension $\sigma _0$ (Fig. 7a). In this section, all the variables are made dimensionless with the needle radius $R_0$, the inertio-capillary time $t_0=(\rho R_0^3/\sigma _0)^{1/2}$, the inertio-capillary velocity $v_0=R_0/t_0$, and the capillary pressure $\sigma _0/R_0$. The velocity $\mathbf{v}(\mathbf{r},t)$ and reduced pressure $p(\mathbf{r},t)$ fields are calculated from the continuity and Navier–Stokes equations

7 $$\begin{aligned}&{\varvec{\nabla }}\cdot \mathbf{v}=0, \end{aligned}$$

8 $$\begin{aligned}&\frac{\partial \mathbf{v}}{\partial t}+\mathbf{v}\cdot {\varvec{\nabla }}\mathbf{v}=-{\varvec{\nabla }}p+{\varvec{\nabla }}\cdot \mathbf{T}, \end{aligned}$$

respectively, where $\mathbf{T}=\text {Oh}[{\varvec{\nabla }}\mathbf{v}+({\varvec{\nabla }}{} \mathbf{v})^T]$ is the viscous stress tensor, and $\text {Oh}=\mu (\rho \sigma _0 R_0)^{-1/2}$ is the volumetric Ohnesorge number. These equations are integrated over the liquid domain of (dimensionless) volume *V* considering the non-slip boundary condition at the solid surface, the anchorage condition at the needle edge, and the kinematic compatibility condition at the free surface.

Neglecting the dynamic effects of the surrounding gas, the balance of normal and tangential stresses at the free surface yields

9 $$\begin{aligned} -p+B\, z+\mathbf{n}\cdot \mathbf{T}\cdot \mathbf{n}=\mathbf{n}\cdot \varvec{\tau }^S, \quad \mathbf{t}\cdot \mathbf{T}\cdot \mathbf{n}=\mathbf{t}\cdot \varvec{\tau }^S, \end{aligned}$$

where $B=\rho g R_0^2/\sigma _0$ is the Bond number, *g* the gravitational acceleration, $\mathbf{n}$ the unit outward normal vector, $\mathbf{t}$ the unit vector tangential to the free surface meridians, and

10 $$\begin{aligned} {\varvec{\tau }}^S=-\mathbf{n}(\varvec{\nabla }^S\cdot \mathbf{n}){\hat{\sigma }}+ \varvec{\nabla }^S{\hat{\sigma }} -\mathbf{n}(\varvec{\nabla }^S\cdot \mathbf{n})\left( \text {Oh}_2^S- \text {Oh}_1^S\right) (\varvec{\nabla }^S\cdot \mathbf{v}) +\varvec{\nabla }^S[\left( \text {Oh}_2^S-\text {Oh}_1^S \right) (\varvec{\nabla }^S\cdot \mathbf{v})]+2\varvec{\nabla }^S\cdot (\text {Oh}_1^S{{\mathbf {\mathsf{{D}}}}}^S), \end{aligned}$$

is the surface stress tensor^41^. Here, ${{\mathbf {\mathsf{{ D}}}}}^S=1/2\, [\varvec{\nabla }^S \mathbf{v}\cdot {{\mathbf {\mathsf{{ I}}}}}^S+{{\mathbf {\mathsf{{I}}}}}^S\cdot (\varvec{\nabla }^S \mathbf{v})^T]$, $\varvec{\nabla }^ S$ is the tangential intrinsic gradient along the free surface, $\mathbf{v}$ the (3D) fluid velocity on the free surface, ${\mathbf {\mathsf{{I}}}}^S$ is the tensor that projects any vector on that surface, ${\widehat{\sigma }}\equiv \sigma /\sigma _0$ is the ratio of the local value $\sigma$ of the surface tension to its equilibrium value $\sigma _0$, $\text {Oh}_{1,2}^S=\mu _{1,2}^S(\rho \sigma _0 R_0^3)^{-1/2}$ are the superficial Ohnesorge numbers defined in terms of the surface shear and dilatational viscosities $\mu _1^S$ and $\mu _2^S$, respectively.

The surface viscosities are expected to depend on the surfactant surface concentration. For the sake of simplicity, we assume the linear relationships $\mu _{1,2}^S=\mu _{1,2}^{S*}{\widehat{\Gamma }}/{\widehat{\Gamma }}_{\text{cmc }}$, where $\mu _{1,2}^{S*}$ are the surfactant viscosities at the cmc. In addition, ${\widehat{\Gamma }}\equiv \Gamma /\Gamma _0$ and ${\widehat{\Gamma }}_{\text{cmc }}\equiv \Gamma _{\text{cmc }}/\Gamma _0$, where $\Gamma$ and $\Gamma _{\text{cmc }}$ are the surfactant surface concentration and its value at the cmc, respectively, both in terms of the equilibrium value $\Gamma _0$. Therefore,

11 $$\begin{aligned} \text {Oh}_{1,2}^S=\text {Oh}_{1,2}^{S*} \frac{{\widehat{\Gamma }}}{{\widehat{\Gamma }}_{\text{cmc }}}, \end{aligned}$$

where $\text {Oh}_{1,2}^{S*}=\mu ^{S*}_{1,2}(\rho \sigma _0 R_0^3)^{-1/2}$ are the superficial Ohnesorge numbers at the cmc.

To calculate the surfactant surface concentration, we take into account that the droplet breakup time is much smaller than the characteristic adsorption–desorption times, and, therefore, surfactant solubility can be neglected over the breakup process. In this case, one must consider the equation governing the surfactant transport on the free surface:

12 $$\begin{aligned} \frac{\partial {\widehat{\Gamma }}}{\partial t}+{\varvec{\nabla }}^S\cdot ({\widehat{\Gamma }}{\mathbf {v}}^S)+{\widehat{\Gamma }}{} \mathbf{n}\cdot ({\varvec{\nabla }}^{S}\cdot \mathbf{n})\mathbf{v}=\frac{1}{\text {Pe}^S}\, {\varvec{\nabla }}^{S2}{\widehat{\Gamma }}, \end{aligned}$$

where Pe$^S$ = $R_0^2/(t_0 {{{\mathcal {D}}}}^S)$ and ${{\mathcal {D}}}^S$ are the surface Peclet number and diffusion coefficient, respectively. The equation of state ${\widehat{\sigma }}({\widehat{\Gamma }})$ is obtained from experimental data as explained below. The free surface becomes saturated for ${\widehat{\Gamma }}\simeq {\widehat{\Gamma }}_{\text{cmc }}$. To reproduce this effect in the simulations, if ${\widehat{\Gamma }}$ exceeds ${\widehat{\Gamma }}_{\text{cmc }}$ at some point and time, we set ${\widehat{\Gamma }}={\widehat{\Gamma }}_{\text{cmc }}$ at that point and time.

### Numerical simulation

The theoretical model is numerically solved by mapping the time-dependent liquid region onto a fixed numerical domain through a coordinate transformation. The transformed spatial domains were discretized using 11 Chebyshev spectral collocation points in the transformed radial direction and 5001 equally spaced collocation points in the transformed axial direction. The axial direction was discretized using fourth-order finite differences. Second-order backward finite differences were used to discretize the time domain^42^. The time step was adapted in the course of the simulation according to the formula $\Delta t=0.025 R_{\text{min }}/v_0$. To deal with the free surface overturning taking place right before the droplet breakup, a quasi-elliptic transformation^43^ was applied to generate the mesh. To trigger the pendant drop breakup process, a very small force was applied to a stable shape with a volume just below the critical one. This perturbation was expected to affect neither the pendant drop dynamics close to the free-surface pinch-off nor the formation of the satellite droplet. The time-dependent mapping of the physical domain does not allow the algorithm to surpass the free surface pinch-off, and therefore the evolution of the satellite droplet cannot be analyzed. The breakup time in the simulation was calculated from the linear extrapolation of the last $N_b=10$ values of $R_{\text{min }}(t)$.

We verified that the results are practically the same for the time interval analyzed in this study when the total number of grid points is doubled (see Supplementary Information). We checked that the value of $N_b$ does not significantly affect the curve $R_{\text{min }}(\tau )$ over the time interval considered in our analysis (see Supplementary Information).

### Experimental method

The experimental method is similar to that used by Rubio et al.^44^ to study the extensional flow of very weakly viscoelastic polymer solutions. In the experimental setup (Fig. 7b), a cylindrical feeding capillary (**A**) $R_0=115$ $\upmu$m in outer radius was placed vertically. To analyze the role of the capillary size, we also conducted experiments with $R_0=205$ $\upmu$m. A pendant droplet was formed by injecting the liquid at a constant flow rate with a syringe pump (Harvard Apparatus PHD 4400) connected to a stepping motor. We used a high-precision orientation system and a translation stage to ensure the correct position and alignment of the feeding capillary. Digital images of the drop were taken using an ultra-high-speed video camera (kirana-5M) (B) equipped with optical lenses (an Optem HR $\times$ 50 magnification zoom-objective and a NAVITAR $\times$ 12 set of lenses) (**C**) (Fig. 7c). As explained below, the images were acquired either at $5 \times 10^6$ fps with a magnification 101.7 nm/pixel or at $5 \times 10^5$ fps with a magnification 156 nm/pixel. The camera could be displaced both horizontally and vertically using a triaxial translation stage (D) with one of its horizontal axes (axis *x*) motorized (THORLABS Z825B) and controlled by the computer, which allowed as to set the droplet-to-camera distance with an error smaller than 29 nm. The camera was illuminated with a laser (SI-LUX 640, specialised imaging) (E) synchronized with the camera, which reduced the effective exposure time down to 100 ns. The camera was triggered by an optical trigger (SI-OT3, specialised imaging) (F), equipped with optical lenses (G) and illuminated with cold white backlight (H). All these elements were mounted on an optical table with a pneumatic anti-vibration isolation system (I) to damp the vibrations coming from the building.

In the experiment, a pendant droplet hanging on the feeding capillary was inflated by injecting the liquid at 1 ml/h. The triple contact lines anchored to the outer edge of the capillary. The drop reached its maximum volume stability limit after around 20 s. We analyzed images of the quasi-static process with the Theoretical Image Fitting Analysis (TIFA)^46^ method to verify that the surface tension right before the droplet breakup was the same (within the experimental uncertainty) as that measured at equilibrium. In this way, one can ensure that the surfactant surface concentration corresponded to the prescribed volumetric concentration at equilibrium. This conclusion can be anticipated from the fact that the characteristic surfactant adsorption process is much smaller than the droplet inflation time.

When the maximum volume stability limit was reached, the droplet broke up spontaneously. We recorded 180 images at $5 \times 10^6$ fps of the final stage of the breakup process within a spatial window $94 \times 78$ $\upmu$m. This experiment was repeated several times to assess the degree of reproducibility of the experimental results. The flow rate at which the pendant droplet is inflated was reduced down to 0.1 ml/h to verify that this parameter did not affect the final stage of the breakup process. Besides, 180 images of a spatial window $144 \times 120$ $\upmu$m were taken at $5 \times 10^5$ fps to describe the process on a larger scale.

We selected SDS in deionized water (DIW) because it is a solution widely used in experiments and very well characterized. The dependence of the surface tension with respect to the surface surfactant concentration $\Gamma$ has been determined from direct measurements (Fig. 7d)^45^. We use the fit

13 $$\begin{aligned} \sigma =10^3\frac{-17.94\,\Gamma +60.76}{\Gamma ^2-240.9\,\Gamma +841.8}, \end{aligned}$$

to that experimental data in our simulations. In this equation, $\sigma$ and $\Gamma$ are measured in mN/m and $\upmu$mol/m$^2$, respectively. It should be noted that there is no theoretical justification for the above equation of state. It simply represents an accurate approximation for the numerical simulations. Other equations may be equally valid for our purposes.

Table 1 shows some physical properties of SDS in DIW. The shear $\mu _1^{S*}$ and dilatational $\mu _2^{S*}$ surface viscosities of aqueous solutions of SDS at the cmc have been widely measured with different methods over the last decades. Zell et al.^6^ reported the surface shear viscosity to be below $10^{-8}$ Pa s m (the sensitivity limit of their technique). Other authors have measured values up to five orders of magnitude higher than that upper bound^47,48^.

**Table 1:** Physical properties of SDS in DIW: superficial viscosities $\mu _{1,2}^{S*}$, surfactant surface diffusivity ${{\mathcal {D}}}^S$, adsorption $t_a$ and desorption $t_d$ time, aggregation number $N_{\text{agg }}$, and micelle radius $R_{\text{mic }}$.

$\mu _1^{S*}$ (Pa s m)^6^	$<10^{-8}$
$\mu _2^{S*}$ (Pa s m)^47^	$10^{-7}$–$10^{-9}$
${{\mathcal {D}}}^S$ (m$^2$/s)^47^	$8 \times 10^{-10}$
$t_a$ (ms)^24^	100
$t_d$ (ms)^47^	169.5
$\Gamma _{\text{cmc }}$ ($\upmu$mol m$^{-2}$)	3.19
$N_{\text{agg }}$^49^	61
$R_{\text{mic }}$ (nm)^49^	1.72

Table 2 shows the values of the superficial Ohnesorge numbers, Boussinesq numbers $\text {Bq}_{1,2}=\mu _{1,2}^S/(\mu \ell _c)$, and surface Peclet number calculated from the values shown in Table 1. The superficial Ohnesorge numbers are much smaller than the volumetric one, $\text {Oh}\simeq 0.02$, which indicates that the superficial viscosities play no significant role on a scale given by the feeding capillary radius $R_0$. The Boussinesq numbers are defined in terms of the characteristic length $\ell _c\equiv 1$ $\upmu$m of the pinching region (see “Results and discussion” section). Due to the smallness of this length, superficial viscous stresses may become comparable with the bulk ones, and, therefore, may produce a measurable effect on that scale. The value of the Peclet number indicates that surfactant surface diffusion is negligible at the beginning of the droplet breakup. The Peclet number defined in terms of $\ell _c$ and the corresponding capillary time $(\rho \ell _c^3/\sigma _0)^{1/2}$ takes values of the order of $10^3$–$10^4$. Therefore, one can expect surface diffusion to play a secondary role on that scale too.

**Table 2:** Dimensionless numbers calculated from the physical properties of SDS in DIW (Table 1): interfacial Ohnesorge numbers Oh$_{1,2}^S$, Boussinesq numbers $\text {Bq}_{1,2}$, and surface Peclet number $\text {Pe}^S$.

Oh$_1^S$	$<9.35\times 10^{-4}$
Oh$_2^S$	$9.35\times 10^{-3}$–$9.35\times 10^{-5}$
$\text {Bq}_1$	$<1.41$
$\text {Bq}_2$	14.1–0.14
$\text {Pe}^S$	$7.73\times 10^{4}$

## Supplementary Information

Supplementary Information.
