# Author Correction: Influence of the surface viscous stress on the pinch-off of free surfaces loaded with nearly-inviscid surfactants

**DOI:** 10.1038/s41598-022-07660-z

**Published:** 2022-04-06

**Authors:** A. Ponce-Torres, M. Rubio, M. A. Herrada, J. Eggers, J. M. Montanero

**Affiliations:** 1 Depto. de Ingeniería Mecánica, Energética y de los Materiales and Instituto de Computación Científica Avanzada (ICCAEx), Universidad de Extremadura, 06006 Badajoz, Spain; 2 Depto. de Mecánica de Fluidos e Ingeniería Aeroespacial, Universidad de Sevilla, 41092 Sevilla, Spain; 3 School of Mathematics, University of Bristol, Fry Building, Bristol, BS8 1UG UK

Correction to: *Scientific Reports* 10.1038/s41598-020-73007-1, published online 30 September 2020

The original version of this Article contained errors.

As pointed out by recent works^1–4^, the velocity appearing in the Boussinesq-Scriven constitutive equation^5^ for the viscous interfacial stress is the 3D fluid velocity evaluated on the interface, including both the tangential and normal components to that surface. Previous studies (see, e.g.,^6–8^) have erroneously interpreted this velocity as the 2D velocity resulting from the projection of the fluid velocity onto the interface, which can lead to significant errors for high interface curvatures. Besides, there are works in which it is not clear whether the velocity in the Boussinesq-Scriven equation was misinterpreted as explained above, or the authors were implicitly assuming that the surface was always flat when that equation was invoked (see, e.g.,^9,10^).

The balance of normal and tangential stresses at the free surface yields.

$$- p + Bz + {\mathbf{n}} \cdot {\mathbf{T}} \cdot {\mathbf{n}} = {\mathbf{n}} \cdot \tau^{s} ,\qquad {\mathbf{t}} \cdot {\mathbf{T}} \cdot {\mathbf{n}} = {\mathbf{t}} \cdot \tau^{s}$$

Due to the above-mentioned misinterpretation of the velocity on the surface, the viscosity contribution to the stress tensor $\tau^{s}$ was not calculated correctly in the paper. This quantity should read^1^

$$\tau^{S} = - \mathbf{n}\left( {\varvec\nabla^{S} \cdot {\mathbf{n}}} \right)\hat{\sigma } + \varvec\nabla^{S} \hat{\sigma } - \mathbf{n}\left( {\varvec\nabla^{S} \cdot {\mathbf{n}}} \right)\left( {{\text{Oh}}_{2}^{S} - {\text{Oh}}_{1}^{S} } \right)\left( {\varvec\nabla^{S} \cdot {\mathbf{v}}} \right) + \varvec\nabla^{S} \left[ {\left( {{\text{Oh}}_{2}^{S} - {\text{Oh}}_{1}^{S} } \right)\left( {\varvec\nabla^{S} \cdot {\mathbf{v}}} \right)} \right] + 2\varvec\nabla^{S} \cdot \left( {{\text{Oh}}_{1}^{S} {\textsf{D}}^{S} } \right),$$

where ${\textsf{D}}^{S} = 1/2([\varvec\nabla^{S} {\mathbf{v}}{ } \cdot { }{\textsf{I}}^{S} + { }{\textsf{I}}^{S} \cdot (\varvec\nabla^{S} {\mathbf{v}})^{T} { }].$ It must be pointed out that ${\mathbf{v}}$ is the (3D) fluid velocity on the free surface.

The above equations replace equations 8 and 10 and due to renumbering are now listed as equations 9 and 10.

As a result of the above, the Methods have been revised. The changes are outlined below.

In the Methods section, under the subheading ‘Theoretical model’,

“The theoretical model is that considered by Ponce-Torres et al.^7^” has now been removed.

In addition, under the subheading ‘Theoretical model’,

“Neglecting the dynamic effects of the surrounding gas, the balance of normal stresses at the free surface yields^44^

8 $$- p + Bz + {\mathbf{n}} \cdot {\mathbf{T}} \cdot {\mathbf{n}} = \left[ {\hat{\sigma } + \left( {{\text{Oh}}_{2}^{S} - {\text{Oh}}_{1}^{S} } \right)\varvec\nabla^{S} \cdot {\mathbf{v}}^{S} } \right]\kappa + 2{\text{Oh}}_{1}^{S} [\kappa_{1} (\varvec\nabla^{S} {\mathbf{v}}^{S} )_{11} + \kappa_{2} \left( {\varvec\nabla^{S} {\mathbf{v}}^{S} )_{22} } \right],$$

where $B = \rho gR_{0}^{2} /\sigma_{0}$ is the Bond number, *g* the gravitational acceleration, ${\mathbf{n}}$ the unit outward normal vector, $\hat{\sigma } \equiv \sigma /\sigma_{0}$ is the ratio of the local value $\sigma$ of the surface tension to its equilibrium value $\sigma_{0},$ ${\text{Oh}}_{1,2}^{S} = \mu_{1,2}^{S} (\rho \sigma_{0} R_{0}^{3} )^{ - 1/2}$ are the superficial Ohnesorge numbers defined in terms of the surface shear and dilatational viscosities $\mu_{1}^{S}$ and $\mu_{2}^{S}$, respectively, $\varvec\nabla^{S}$ the tangential intrinsic gradient along the free surface, ${\mathbf{v}}^{S} \left( {z,t} \right)$ the (two-dimensional) tangential velocity to the free surface, $\kappa = \kappa_{1} + \kappa_{2}$ (twice) the mean curvature of the free surface, $\kappa_{1}$ and $\kappa_{2}$ the curvatures along the meridians and parallels in the inward normal direction, respectively, and $(\varvec\nabla^{S} {\mathbf{v}}^{S} )_{11}$ and $(\varvec\nabla^{S} {\mathbf{v}}^{S} )_{22}$ the diagonal elements of $\varvec\nabla^{S} {\mathbf{v}}^{S}$ along the meridians and the parallels, respectively.

In addition, the balance of tangential stresses leads to

9 $${\mathbf{t}} \cdot {\mathbf{T}} \cdot {\mathbf{n}} = {\mathbf{t}} \cdot \varvec\tau^{S} ,$$

where ${\mathbf{t}}$ is the unit vector tangential to the free surface meridians, and

10 $$\varvec\tau^{S} = \varvec\nabla^{S} \hat{\sigma } + \varvec\nabla^{S} \cdot \{ {\text{Oh}}_{1}^{S} [\varvec\nabla^{S} {\mathbf{v}}^{S} + \left( {\varvec\nabla^{S} {\mathbf{v}}^{S} )^{{ \top }} } \right]\} + \varvec\nabla^{S} \left[ {\left( {{\text{Oh}}_{2}^{S} - {\text{Oh}}_{1}^{S} } \right)\varvec\nabla^{S} \cdot {\mathbf{v}}^{S} } \right],$$

is the surface stress tensor.”

now reads:

“Neglecting the dynamic effects of the surrounding gas, the balance of normal stresses at the free surface yields.

9 $$- p + Bz + {\mathbf{n}} \cdot {\mathbf{T}} \cdot {\mathbf{n}} = {\mathbf{n}} \cdot \varvec\tau^{S} ,\qquad {\mathbf{t}} \cdot {\mathbf{T}} \cdot {\mathbf{n}} = {\mathbf{t}} \cdot \varvec\tau^{S},$$

where $B = \rho gR_{0}^{2} /\sigma_{0}$ is the Bond number, *g* the gravitational acceleration, ${\mathbf{n}}$ the unit outward normal vector, ${\mathbf{t}}$ the unit vector tangenital to the free surface meridians, and

10 $$\varvec\tau^{S} = - {\mathbf{n}}\left( {\varvec\nabla^{S} \cdot {\mathbf{n}}} \right)\hat{\sigma } + \varvec\nabla^{S} \hat{\sigma } - {\mathbf{n}}\left( {\varvec\nabla^{S} \cdot {\mathbf{n}}} \right)\left( {{\text{Oh}}_{2}^{S} - {\text{Oh}}_{1}^{S} } \right)\left( {\varvec\nabla^{S} \cdot {\mathbf{v}}} \right) + \varvec\nabla^{S} \left[ {\left( {{\text{Oh}}_{2}^{S} - {\text{Oh}}_{1}^{S} } \right)\left( {\varvec\nabla^{S} \cdot {\mathbf{v}}} \right)} \right] + 2\varvec\nabla^{S} \cdot \left( {{\text{Oh}}_{1}^{S} {\textsf{D}}^{S} } \right),$$

is the surface stress tensor^41^. Here, ${\textsf{D}}^{S} = 1/2([\varvec\nabla^{S} {\mathbf{v}}{ } \cdot { }{\textsf{I}}^{S} + { }{\textsf{I}}^{S} \cdot (\varvec\nabla^{S} {\mathbf{v}})^{T} { }],{ }\varvec\nabla^{S}$ is the tangential intrinsic gradient along the free surface, ${\mathbf{v}}$ the (3D) fluid velocity on the free surface, ${\textsf{I}}^{S}$ is the tensor that projects any vector on that surface, $\hat{\sigma } \equiv \sigma /\sigma_{0}$ is the ratio of the local value $\sigma$ of the surface tension to its equilibrium value $\sigma_{0}$, ${\text{Oh}}_{1,2}^{S} = \mu_{1,2}^{S} (\rho \sigma_{0} R_{0}^{3} )^{ - 1/2}$ are the superficial Ohnesorge numbers defined in terms of the surface shear dilatational viscosities $\mu_{1}^{S}$ and $\mu_{2}^{S}$, respectively.”

As a result of the above, the numerical results have been re-calculated and the Results and discussion section revised. Consequently, Figure 3, Figure 4, Figure 6 and Figure 7 and their associated legends contained errors and have now been replaced.

Using the corrected expression for the surface viscous stresses, the dilatational viscosity has been found to play a negligible role. Besides, the (2D) velocity on the surface makes no sense anymore. For those reasons, the original Figure 5 of the paper no longer applies and has subsequently been removed.

The original Figures 3, 4, 5, 6 and 7 and their accompanying legends appear below.

**Figure 3:**
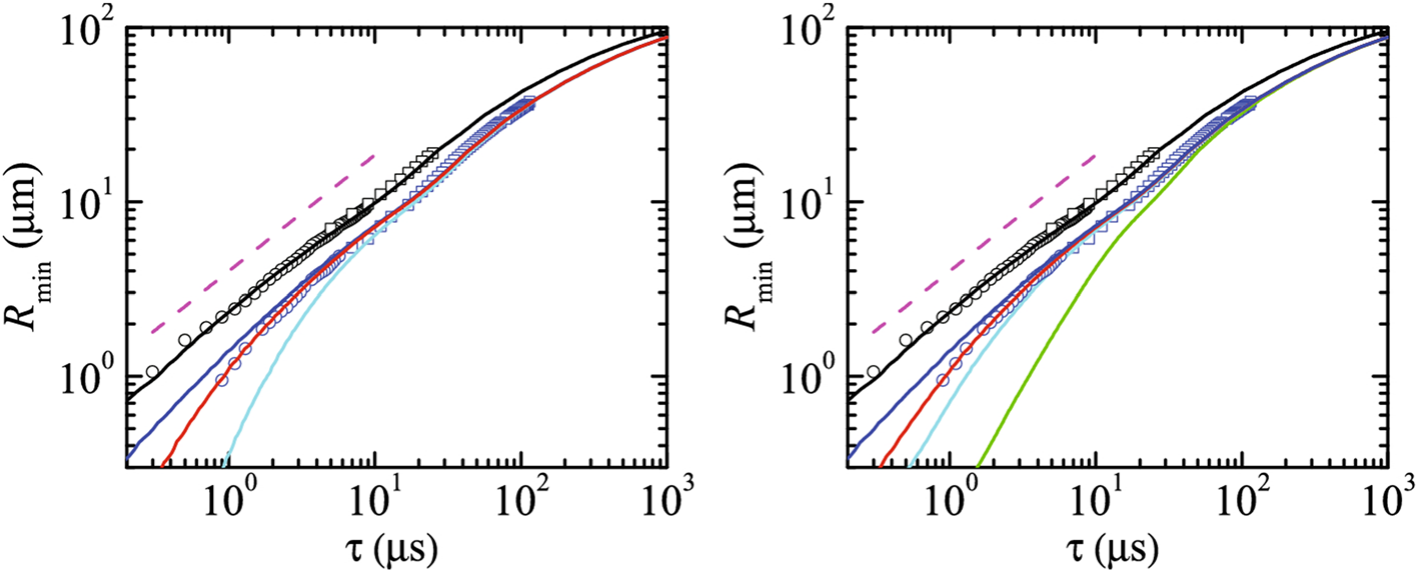
$R_{\text{min }}(\tau )$ or the breakup of a pendant drop of DIW and DIW+SDS 0.8cmc. The black and blue symbols are the experimental data for DIW and DIW+SDS 0.8cmc, respectively. The different symbols correspond to experiments visualized with different magnifications. The black solid line and magenta dashed line correspond to the simulation and the power law $R_{\text{min }}(\tau )\sim \tau ^{2/3}$ for DIW, respectively. (Left) The colored solid lines correspond to simulations of DIW+SDS 0.8cmc for $\mu _1^{S*}=0$ and $\mu _2^{S*}=0$ (blue), $5 \times 10^{-10}$ (red), and $3.5 \times 10^{-9}$ Pa s m (cyan). (Right) the colored solid lines correspond to simulations of DIW+SDS 0.8cmc for $\mu _1^{S*}=0$ and $\mu _2^{S*}=0$ (blue), $3.5 \times 10^{-9}$ (red) $10^{-8}$ (cyan), and  $10^{-7}$ Pa s m (green). All the numerical results were calculated for $B=3.396 \times 10^{-3}$, $\text {Oh}=0.01510$, ${\widehat{\Gamma }}_{\text{cmc }}=1.002$, and Pe$^S=7.730 \times 10^{4}$ (see “Methods” section). In the left-hand graph, the colored solid lines correspond to Oh$_2^{S*}=0$ and Oh$_1^{S*}=0$ (blue), $6.563 \times 10^{-5}$ (red), and $4.594 \times 10^{-4}$ (cyan). In the right-hand graph, the colored solid lines correspond to Oh$_1^{S*}=0$ and Oh$_2^{S*}=0$ (blue), $4.594 \times 10^{-4}$ (red), $1.313 \times 10^{-3}$ (cyan), and $1.313 \times 10^{-2}$ (green).

**Figure 4:**
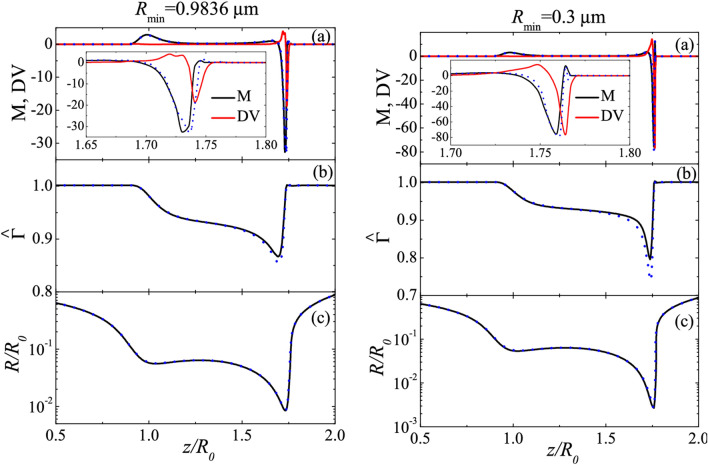
Axial distribution of the Marangoni stress (M) and tangential dilatational viscous stress (DV) (**a**), surfactant surface concentration (**b**), and free surface radius (**c**) for DIW+SDS 0.8cmc. The solid lines are the results for $$\{$$$${\mu _1^{S*}= 0}$$, $$\mu _2^{S*}= 3.5 \times 10^{-9}$$ Pa s m$$\}$$, while the dotted lines correspond to $$\mu _1^{S*}=\mu _2^{S*}=0$$ (in the right-hand graphs, $$R_{\text{min }}=0.3$$ $$\upmu$$m for $$\mu _1^{S*}=\mu _2^{S*}=0$$. The results were calculated for $$B=3.396 \times 10^{-3}$$, Oh = 0.0151, $${\widehat{\Gamma }}_{\text{cmc }}=1.002$$, Pe$$^S=7.730 \times 10^{4}$$, Oh$$_1^{S*}=0$$, and Oh$$_2^{S*}=4.594 \times 10^{-4}$$ (solid lines) and 0 (dotted lines) (see “Methods” section).

**Figure 5:**
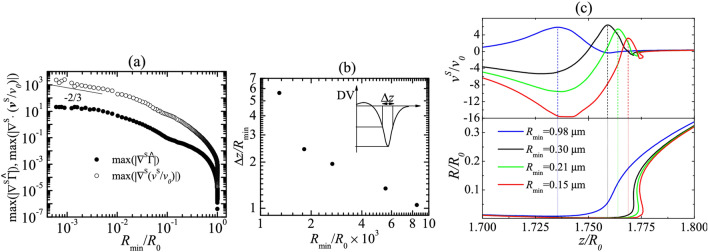
Maximum values of the surfactant gradient, $\text {max} (| \varvec\nabla ^S \widehat{\Gamma } | )$ (solid symbols), and the surface velocity gradient, $\text {max} \left( \left| \varvec\nabla ^S \cdot \mathbf{v} ^S \right| \right)$ (open symbols). (**b**) Full width at half maximum, Δ*z* of the dilatational viscous stress as a function of the minimum radius $R_{\text{min}}$. (**c**) Surface velocity $\nu ^S (z)$ (upper graph) and free surface radius $R(z)$ (lower graph). The dashed vertical lines indicate the position of the free surface neck. In all the cases, the results were calculated for DIW+SDS 0.8cmc with {${\mu _1^{S*}= 0}$, $\mu _2^{S*}= 3.5 \times 10^{-9}$ Pa s m$\}$. The values of the dimensionless parameters are $B=3.396 \times 10^{-3}$, Oh = 0.0151, ${\widehat{\Gamma }}_{\text{cmc }}=1.002$, Pe$^S=7.730 \times 10^{4}$, Oh$_1^{S*}=0$, and Oh$_2^{S*}=4.594 \times 10^{-4}$(see “Methods” section).

**Figure 6:**
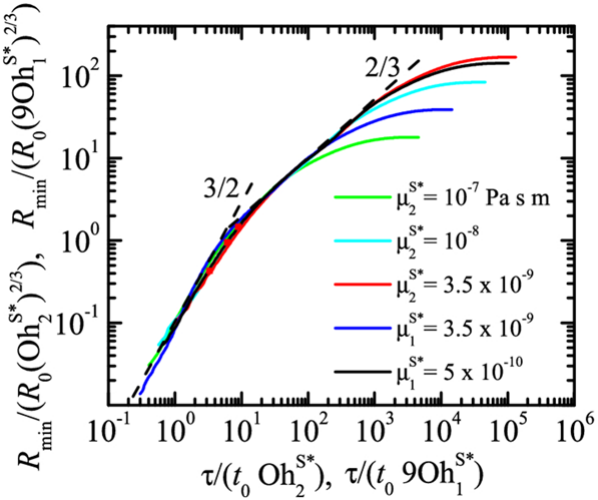
Dimensionless minimum radius $R_{\text{min }}/R_0$ as a function of the dimensionless time to the breakup, $\tau /t_0$, or the breakup of a pendant drop of DIW+SDS 0.8cmc. The labels indicate the values of the non-zero shear/dilatational viscosity in each case. The results were calculated for $B=3.396 \times 10^{-3}$, Oh = 0.01510, ${\widehat{\Gamma }}_{\text{cmc }}=1.0016$, and Pe$^S=7.73 \times 10^{4}$ (see “Methods” section).

**Figure 7:**
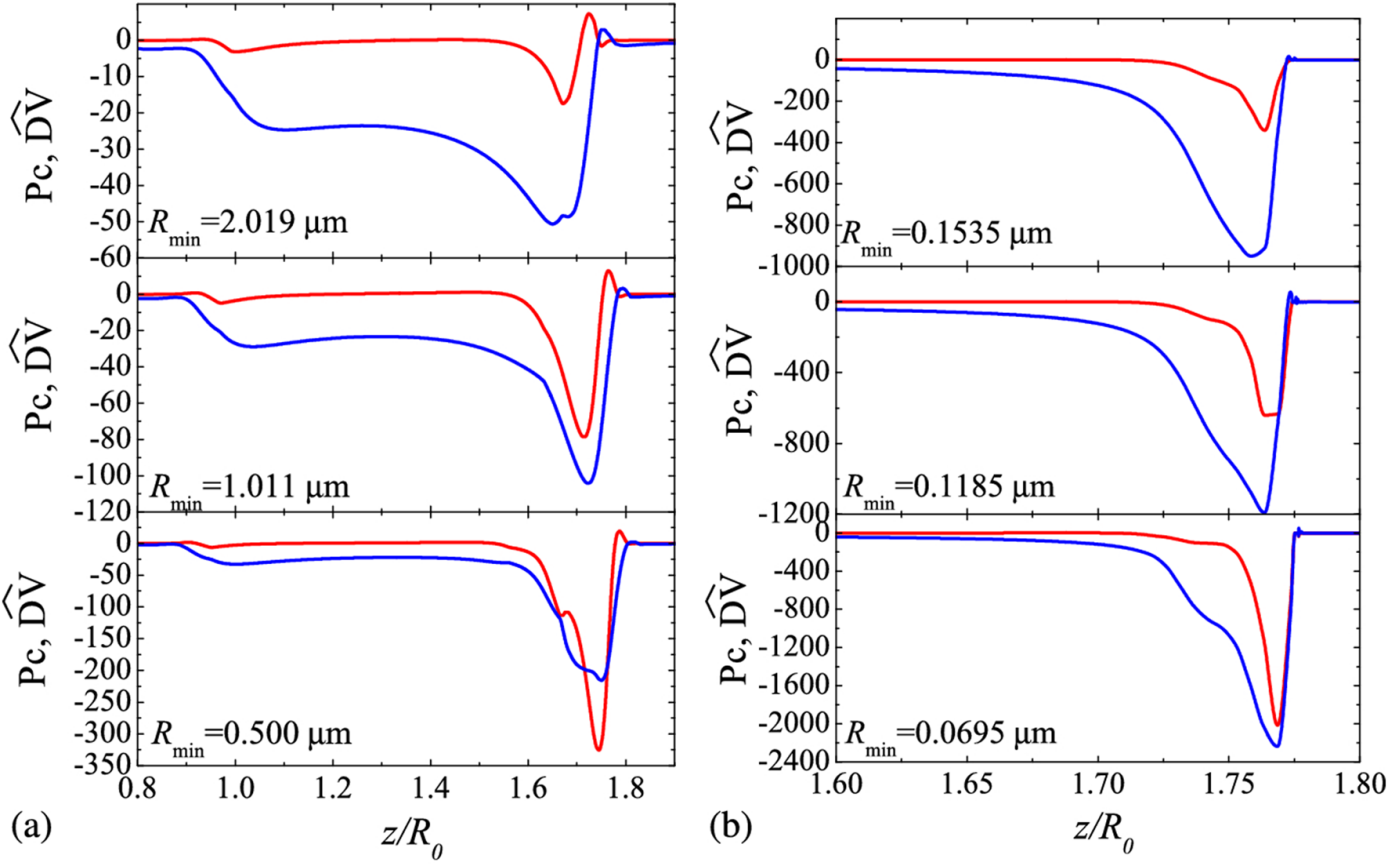
Axial distribution of the capillary stress $\text{Pc}=\hat \sigma \kappa$ (blue lines) and normal dilatational viscous stress $\widehat{\text {DV}}=\text {Oh}_2^S(\varvec{\nabla }^S\cdot \mathbf {v}^S) \kappa$ (red lines) for DIW+SDS 0.8cmc and three instants as indicated by the value of $R_{\text{min}}$. The left-hand and right-hand graphs correspond to {${\mu _1^{S*}= 0}$, $\mu _2^{S*}= 10^{-7}$ Pa s m$\}$ and ${\mu _1^{S*}= 0}$, $\mu _2^{S*}= 3.5 \times 10^{-9}$ Pa s m$\}$, respectively. The results were calculated for $B=3.396 \times 10^{-3}$, Oh = 0.01510, ${\widehat{\Gamma }}_{\text{cmc }}=1.002$, and Pe$^S=7.730 \times 10^{4}$, Oh$_1^{S*}=0$, and Oh$_2^{S*}=1.313 \times 10^{-2}$ (left-hand graphs) and $4.594 \times 10^{-4}$ (right-hand graphs) (see “Methods” section).

As a result of the changes, the numbering of Figures has been revised.

The fitting values for the surface viscosities have been re-calculated.

In the Results and discussion section, 5^th^ paragraph.

“The experimental results can be reproduced for $\mu_{1}^{S*} = 5 \times 10^{ - 10}$ Pa s m and $\mu_{2}^{S*} = 0$ (Fig. 3-left). This upper bound of the surface shear viscosity is consistent with the results obtained by Zell et al.^6^, who concluded that the surface shear viscosity of SDS in DIW must take values below 10^-8^ Pa s m (the sensitivity limit of their technique). The experimental results can also be reproduced for $\mu_{1}^{S*} = 0$ and $\mu_{2}^{S*} = 3.5 \times 10^{ - 9}$ Pa s m (Fig. 3-right). There are significant deviations when other values of $\mu_{2}^{S*}$ found in the literature are considered^36^. The optimum value of the shear viscosity is one order of magnitude smaller than that of the dilatational viscosity, which suggests that shear viscous stresses have a greater effect on the pinching than dilatational ones for the same value of the corresponding surface viscosities. In fact, when the surface shear viscosity takes the value of the dilatational viscosity ($\mu_{1}^{S*} = 3.5 \times 10^{ - 9 }$ Pa s m, $\mu_{2}^{S*} = 0$) the numerical curve (cyan solid line in Fig. 3-left) significantly deviates from the experimental one. The relative importance of the shear and dilatational viscosities can be explained in terms of the equivalence between the corresponding terms in the 1D approximation, as will be discussed below. Similar conclusions can be drawn from the experiments with DIW+SDS 2cmc (see Supplementary Information).”

now reads:

“The numerical results fit the experimental measurements for $\mu_{1}^{S*} = 1.2 \times 10^{ - 9}$ Pa s m and $\mu_{2}^{S*} = 0$ (Fig. 3-left) or $\mu_{1}^{S*} =$ 0 and $\mu_{2}^{S*} = 6 \times 10^{ - 7 }$ Pa s m (Fig. 3-right). As can be observed, the optimum value of the dilatational viscosity $\mu_{2}^{S*}$ is more than two orders of magnitude larger than that of the shear viscosity $\mu_{1}^{S*}$. This means that the effect of the dilatational viscosity is much smaller than that of the shear viscosity. If one assumes that the values of both viscosities are commensurate with each other, the dilatational viscosity plays a negligible role in the filament thinning. This result has practical consequences because it means that the breakup of a pendant drop can be used to measure the shear surface viscosity of a nearly-inviscid surfactant monolayer. The value $\mu_{1}^{S*} = 1.2 \times 10^{ - 9 }$ Pa s m is consistent with the results obtained by Zell et al.^6^, who concluded that the shear viscosity of SDS in DIW must take values below 10^–8^ Pa s m (the sensitivity limit of their technique).

In the Results and discussion section, 6^th^ paragraph,

“When surface viscosities are accounted for, a competition arises between the Marangoni stress, ${\text{M}} \equiv {\mathbf{t}} \cdot \varvec\nabla^{S} \hat{\sigma }$ and the tangential projection of the surface viscous stress,

2 $${\text{SV}} \equiv {\mathbf{t}} \cdot \left[{\varvec\nabla^{S} \cdot \{ \text{Oh}_{1}^{S} [\varvec\nabla^{S} \mathbf{v}^{S} + { (\varvec\nabla^{S} \mathbf{v}^{S} })^{ \top }  ]\} - \varvec\nabla^{S} \left( {{\text{Oh}_{1}^{S} \varvec\nabla^{S} \cdot {\mathbf{v}}^{S} } }\right) }\right],$$

3 $${\text{DV}} \equiv {\mathbf{t}} \cdot \left[ {\varvec\nabla^{S} \left( {{\text{Oh}}_{2}^{S} \varvec\nabla^{S} \cdot {\mathbf{v}}^{S} } \right)} \right],$$

where SV and DV are the (dimensionless) contributions associated with the shear and dilatational surface viscosities, respectively, and ${\text{Oh}}_{1,2}^{S} = \mu_{1,2}^{S} (\rho \sigma_{0} R_{0}^{3} )^{ - 1/2}$ are the superficial Ohnesorge numbers defined in terms of the surface shear and dilatational viscosities $\mu_{1}^{S}$ and $\mu_{2}^{S}$ the liquid density ρρ and equilibrium surface tension $\sigma_{0}$(see “Methods” section). Figure 4 shows the axial distribution of the tangential stresses, surfactant surface concentration, and free surface radius at a given instant of the droplet evolution. We analyze the solution for $\mu_{1}^{S*} = 0$ and the optimum value of the dilatational surface viscosity determined from Fig. 3-right, $\mu_{2}^{S*} = 3.5 \times 10^{ - 9}$ Pa s m (the same comparison is presented in the Supplemental Information but for $\mu_{1}^{S*} = \mu_{2}^{S*} = 0$ and the optimum value of the shear surface viscosity determined from Fig. 3-left, $\mu_{1}^{S*} = 5 \times 10^{ - 10}$  Pa s m). The instants were selected so that $R_{{{\text{min}}}}$ took approximately the same value in the simulations with and without surface viscosities. For $R_{{{\text{min}}}} = 0.9836$ µm, the shear viscous stress is much smaller than the Marangoni stress over the entire free surface. As the minimum radius decreases, the relative importance of the shear viscosity increases. In fact, the maximum value of the shear viscous stress becomes comparable to that of the Marangoni stress for $R_{{{\text{min}}}} = 0.3$ µm. Small differences in the surfactant distribution arise for $R_{{{\text{min}}}} \lesssim 0.3$ *μ*m. The presence of shear viscosity slightly reduces the magnitude of the Marangoni stress.”

now reads:

“Figure 4 shows the values of the axial distribution of the Marangoni stress M and tangenital shear viscous stress SV, the surfactant surface concentration ${\hat{\Gamma }}$, and the free surface radius $R/R_{0}$ for DIW + SDS 0.8cmc. Here,

2 $${\text{M}} \equiv {\mathbf{t}} \cdot \varvec\nabla^{S} \hat{\sigma },\quad {\text{SV}} \equiv {\mathbf{t}} \cdot \{ \varvec\nabla^{S} \cdot {[ - \text{Oh}}_{1}^{S} \left[ {\varvec\nabla^{S} {\mathbf{v}}^{S} } \right){]} + 2\varvec\nabla^{S} \cdot \left( {{\text{Oh}}_{1}^{S} {\textsf{D}}^{S} } \right) ]\}$$

where ${\text{Oh}}_{1}^{S}$ is the superficial Ohnesorge number defined in terms of the surface shear viscosity (see Methods section). The relative importance of the shear viscosity increases as the minimum radius decreases. The presence of shear viscosity slightly reduces the magnitude of the Marangoni stress. The viscous surface stress hardly alters the surfactant distribution and the free surface shape.”

In the Results and discussion section, due to the removal of Figure 5, the following text was removed from paragraph 7:

“While the gradient of surfactant concentration remains bounded in the pinching region, the gradient of surface velocity continues to increase there (Fig. 5a). This may explain why surface viscous stresses grow faster than Marangoni stress over the time interval analyzed. Similar conclusions can be drawn from the numerical simulation conducted for $\{ \mu_{1}^{S*} = 5 \times 10^{ - 10},$ $\mu_{2}^{S*} = 0$ Pa s m} (see Supplementary Information).”

For consistency with the new figures, in the Results and discussion section, 8^th^ paragraph,

“For instance, $R_{{{\text{min}}}} = 0.32$ and 0.57 μm at $\tau \simeq 0.35$ μs for $\{ \mu_{1}^{S*} = 0$ Pa s m, $\mu_{2}^{S*} = 3.5 \times 10^{ - 9} \}$ and $u_{1}^{S*} = \mu_{2}^{S*} = 0$ respectively. However, the free surface shapes are practically the same if they are compared when the same value $R_{{{\text{min}}}} = 0.32$ μm of the minimum radius is reached.”

now reads:

“For instance, $R_{{{\text{min}}}} = 0.24$ and 0.42 μm at $\tau \simeq 0.25$ μs for $\{ \mu_{1}^{S*} = 1.2 \times 10^{ - 9}$ Pa s m, $\mu_{2}^{S*} = 0\}$ and $u_{1}^{S*} = \mu_{2}^{S*} = 0$ respectively. However, the free surface shapes are practically the same if they are compared when the same value $R_{{{\text{min}}}} = 0.24$ μm of the minimum radius is reached.”

In the Results and discussion section, due to the removal of Figure 5, paragraph 9 was removed. This paragraph previously read:

“The dilatational viscous stress exhibits a noticeable maximum near the free surface neck. The full width at half maximum, ${\Delta }z$, measured in terms of the minimum radius, $R_{{{\text{min}}}} ,$ sharply increases as the droplet approaches its breakup (Fig. 5b), which shows the growing importance of the dilatational viscous stress with time. Figure 5c shows the velocity $v^{S}$ (see “Methods” section) along the free surface as the droplet approaches its breakup for the case $\{ \mu_{1}^{S*} = 0$ $\mu_{2}^{S*} = 3.5 \times 10^{ - 9}$ Pa s m}. As can be observed, the maximum of $v^{S} \left( z \right)$, exhibits a non-monotonic behavior with respect to the time to the pinching, and is located at the free surface neck. The difference between the maximum and minimum values of $v^{S} \left( z \right)$  increases with time, and so does the average dilatational stress in the pinching region. The overturning of the free surface is observed for $R_{{{\text{min}}}} \lesssim 0.3$. For this reason, $v^{S} \left( z \right)$, becomes a multivalued function on the right side of the free surface neck.”

In the paper, the authors studied how the scaling of the minimum radius depends on the surfactant viscosities. According to the corrected results, that scaling should be modified.

In the Results and discussion section, 11^th^ paragraph,

“The value of the exponent *β* can be guessed from the balance of forces. Both Marangoni and surface viscous stresses delay the free surface pinch-off (Fig. 3) acting against the driving capillary force. For sufficiently small values of $R_{{{\text{min}}}}$, the effect of surface viscous stresses become comparable and even larger than that caused by Marangoni stress (Fig. 4). The value of $R_{{{\text{min}}}}$ below which this occurs decreases as the surface viscosities decrease. For instance, Marangoni and surface viscous stresses produce similar effects for $R_{{{\text{min}}}} \lesssim 2$ µm and $R_{{{\text{min}}}} \lesssim 0.15$ µm in the cases $\{ \mu_{1}^{S*} = 0,$ $\mu_{2}^{S*} = 10^{ - 7}$ Pa s m} and $\{ \mu_{1}^{S*} = 0,$ $\mu_{2}^{S*} = 3.5 \times 10^{ - 9}$ Pa s m} respectively. Therefore, we expect surface viscous stresses to be commensurate with the driving capillary pressure in the pinch-off region for those intervals of $R_{{{\text{min}}}}$. In fact, the interfacial Ohnesorge numbers defined in terms of $R_{{{\text{min}}}}$ take values at least of order of unity in those intervals.”

now reads, current paragraph 10:

“The value of the exponent *β* can be guessed from the balance of forces. Both Marangoni and surface viscous stresses delay the free surface pinch-off (Fig. 3) acting against the driving capillary force. For sufficiently small values of $R_{{{\text{min}}}}$, the effect of surface viscous stresses become comparable to that caused by Marangoni stress (Fig. 4). The value of $R_{{{\text{min}}}}$ below which this occurs decreases as the surface viscosities decrease. Therefore, we expect surface viscous stresses to be commensurate with the driving capillary pressure in the pinch-off region for $R_{\min } \to 0.$”

In the Results and discussion section, the 12^th^ paragraph was revised to include a new Equation 5.

“The balance between the capillary pressure and the surface viscous stresses in Eq. (8) yields $\sigma_{0} /R_{s} \sim \mu_{1,2}^{S*} /\left( {R_{s} \tau_{s} } \right)$, where we have taken into account that the variation of surface velocity scales as $\left( {R_{s} /\tau_{s} } \right)/R_{s}$ due to the continuity equation. The above balance allows us to conclude that $\beta = 1$ and therefore $\alpha = 2/3$. According to our analysis,

5 $$\begin{array}{*{20}c} {\frac{{R_{\min } }}{{(\mu_{1,2}^{S*} )^{2/3} }} \sim \left( {\frac{\tau }{{\mu_{1,2}^{S*} }}} \right)^{\gamma } } \\ \end{array}$$

in the viscous regime.”

now reads, current paragraph 11:

“The balance between the capillary pressure and the normal surface viscous stresses in Eq. (10) yields $\sigma_{0} /R_{s} \sim \mu_{1,2}^{S*} /\left( {R_{s} \tau_{s} } \right)$, where we have taken into account that the variation of surface velocity scales as $\left( {R_{s} /\tau_{s} } \right)/R_{s}$ due to the continuity equation. The above balance allows us to conclude that $\beta = 1$ and therefore $\alpha = 2/3$. According to our analysis,

4 $$\begin{array}{*{20}c} {\frac{{R_{\min } }}{{(\mu_{1,2}^{S*} )^{2/3} }} \sim \left( {\frac{\tau }{{\mu_{1,2}^{S*} }}} \right)^{\gamma } } \\ \end{array}$$

in the viscous regime. According to our previous results (Fig. 3), we can assume that the dilatational viscosity plays a negligible role. Then, we have

5 $$\begin{array}{*{20}c} {\frac{{R_{\min } }}{{(\mu_{1}^{S*} )^{2/3} }} \sim \left( {\frac{\tau }{{\mu_{1}^{S*} }}} \right)^{\gamma } } \\ \end{array}$$

in surface viscosity-dominated regime. Figure 5 shows the results scaled with those exponents. The simulations show the transition from the inertio-capillary regime $R_{{{\text{min}}}} \sim \tau^{2/3}$ to the asymptotic behavior given by power law $\gamma = 1$. The asymptotic behavior $R_{{{\text{min}}}} \sim \tau$ coincides with that recently derived by Wee et al.^36^ .”

In the Results and discussion section, paragraphs 13, 14 and 15 were removed. These paragraphs previously read:

“In the 1D (slenderness) approximation^37^, the axial forces per unit volume due to the shear and dilatational surface viscosities are $(9\mu_{1}^{S} Rw_{z} )_{z} /2R^{2}$ and $(\mu_{2}^{S} Rw_{z} )_{z} /2R^{2}$ [38], respectively, where *R* is the free surface radius, *w* is the *z*-component of the velocity, and the subscript *z* indicates the derivative with respect to the coordinate *z*. As can be seen, the terms corresponding to the shear and dilatational viscosities differ only by a factor 9. Therefore, the asymptotic behavior of $R_{{{\text{min}}}} \left( \tau \right)$ for $\left\{ {\mu_{1}^{S*} = a,\mu_{2}^{S*} = 0} \right\}$ (*a* is an arbitrary constant) is expected to be the same as that for $\{ \mu_{1}^{S*} = 0,\mu_{2}^{S*} = 9a$}. As will be seen below, this allows us to group the simulation results for $\mu_{1}^{S*} \ne 0$ and $\mu_{2}^{S*} \ne 0$.

Using the equivalence $9\mu_{1}^{S} \leftrightarrow \mu_{2}^{S}$, we find the values of the exponents $\beta$ and $\gamma$ leading to the collapse of all the numerical data for $R_{{{\text{min}}}} \to 0$. Following the optimization method described by Montanero and Gañán-Calvo^39^, the best collapse is obtained for $\beta = 1.1$ and $\gamma = 1.4$. Figure 6 shows the results scaled with the exponents $\beta = 1$ and $\alpha =2/3$ calculated in the previous analysis. As explained above, we have grouped the results for nonzero shear and dilatational viscosities using the factor 9 suggested by the 1D model. The simulations show the transition from the inertio-capillary regime $R_{{{\text{min}}}} \sim \tau^{2/3}$ to the asymptotic behavior given by power law $\gamma = 3/2$.

The axial distributions of the capillary pressure and the dilatational viscous stress are shown in Fig. 7 for the cases $\{ \mu_{1}^{S*} = 0,\mu_{2}^{S*} = 10^{ - 7}$ Pa s m} and $\{ \mu_{1}^{S*} = 0, \mu_{2}^{S*} = 3.5 \times 10^{ - 9}$ Pa s m}. As can be observed, the dilatational viscous stress becomes comparable with the driving capillary pressure for $R_{{{\text{min}}}} \lesssim 2$ and $R_{{{\text{min}}}} \lesssim 0.15$ µm in the cases $\mu_{2}^{S*} = 10^{ - 7}$ Pa s m and $\mu_{2}^{S*} = 3.5 \times 10^{ - 9}$ Pa s m, respectively. This explains the good agreement between the numerical simulations and the scaling proposed above for the minimum radius.”

Paragraph 12 now reads:

“Figure 6 shows the axial distribution of the capillary pressure Pc and normal shear viscous stress $\widehat{{{\text{SV}}}}$ for DIW + SDS 0.8cmc at three instants as indicated by the value of $R_{{{\text{min}}}}$. Here,

6 $${\text{Pc}} = - \left( {\varvec\nabla^{S} \cdot {\mathbf{n}}} \right)\widehat{\sigma}, \quad \widehat{{{\text{SV}}}} = {\text{Oh}}_{1}^{S} \left( {\varvec\nabla^{S} \cdot {\mathbf{n}}} \right)\left( {\varvec\nabla^{S} \cdot {\mathbf{v}}} \right).$$

We consider the shear viscous stress $\widehat{{{\text{SV}}}}$ because the results indicate that shear viscosity plays a more significant role than the dilatational one. The normal shear viscous stress becomes comparable with the capillary pressure as $R_{{{\text{min}}}} \to 0.$”

Lastly, the final paragraph of the Results and discussion section has been revised:

“The pinching of an interface is a singular phenomenon that allows us to test theoretical models under extreme conditions. The vanishing spatiotemporal scales reached by the system as the interface approaches its breakup unveil physical effects hidden in phenomena occurring on much larger scales. This work is an example of this. Surface viscous stresses become relevant in the vicinity of the pinching region long before thermal fluctuations become significant^41,42^, even for practically inviscid surfactants, such as SDS. In this sense, the surfactant-laden pendant droplet can be seen as a very sensitive surfactometer to determine the values of the surface viscosities, which constitutes a difficult problem^43^. A series of experiments for different surfactant concentrations and needle radii may lead to accurate measurements of $\mu_{1}^{S} \left( {\Gamma } \right)$ and $\mu_{2}^{S} \left( {\Gamma } \right)$ characterizing the behavior of low-viscosity surfactants.”

now reads:

“The pinching of an interface is a singular phenomenon that allows us to test theoretical models under extreme conditions. The vanishing spatiotemporal scales reached by the system as the interface approaches its breakup unveil physical effects hidden in phenomena occurring on much larger scales. This work is an example of this. Surface viscous stresses become relevant in the vicinity of the pinching region long before thermal fluctuations become significant^38,39,^ even for practically inviscid surfactants, such as SDS. Besides, the effect of the dilatational surface viscosity on the thinning has shown to be negligible with respect to the shear viscosity. In this sense, the surfactant-laden pendant droplet can be seen as a very sensitive surfactometer to determine the values of the surface shear viscosity, which constitutes a difficult problem^40^. A series of experiments for different surfactant concentrations and needle radii may lead to accurate measurements of $\mu_{1}^{S} \left( {\Gamma } \right)$ characterizing the behavior of low-viscosity surfactants.”

The original Article has been corrected.
