# Supplementary material for: Influence of the surface viscous stress on the pinch-off of free surfaces loaded with nearly-inviscid surfactants
Source: Sci Rep. 2020 Sep 30;10:16065. doi: 10.1038/s41598-020-73007-1 (PMC7528013; doi:10.1038/s41598-020-73007-1)
Supplement: Supplementary file 1 — Supplementary Information. [file 41598_2020_73007_MOESM1_ESM.pdf]

Suplementary information for: Influence of the surface viscous stress on the pinch-off of free surfaces loaded with nearly-inviscid surfactants

A. Ponce-Torres[1], M. Rubio[1], M. A. Herrada[2], J. Eggers[3], and J. M. Montanero[1,*]

[1]Depto. de Ingeniería Mecánica, Energética y de los Materiales and Instituto de Computación Científica Avanzada (ICCAEx), Universidad de Extremadura, E-06006 Badajoz, Spain
[2]Depto. de Mecánica de Fluidos e Ingeniería Aeroespacial, Universidad de Sevilla, E-41092 Sevilla, Spain
[3]School of Mathematics, University of Bristol, Fry Building, Bristol BS8 1UG, UK
[*]jmm@unex.es

Experimental results for DIW+SDS 2cmc can also be reproduced for $\mu_1^{S*} = 5 \times 10^{-10}$ Pa s m and $\mu_2^{S*} = 0$ (see Fig. 1-left) and for $\mu_1^{S*} = 0$ and $\mu_2^{S*} = 3.5 \times 10^{-9}$ Pa s m (see Fig. 1-right). The agreement achieved for DIW+SDS 2cmc is slightly worse than that obtained for DIW+SDS 0.8cmc probably because the experimental surface tension values are less accurate for concentrations larger than the critical micelle concentration (cmc).

In Fig. 2, we compare the solution for $\mu_1^{S*} = \mu_2^{S*} = 0$ and the optimum value of the shear surface viscosity $\mu_1^{S*} = 5 \times 10^{-10}$ Pa s m. The instants were selected so that $R_{\min}$ took approximately the same value in the simulations with and without surface viscosities.

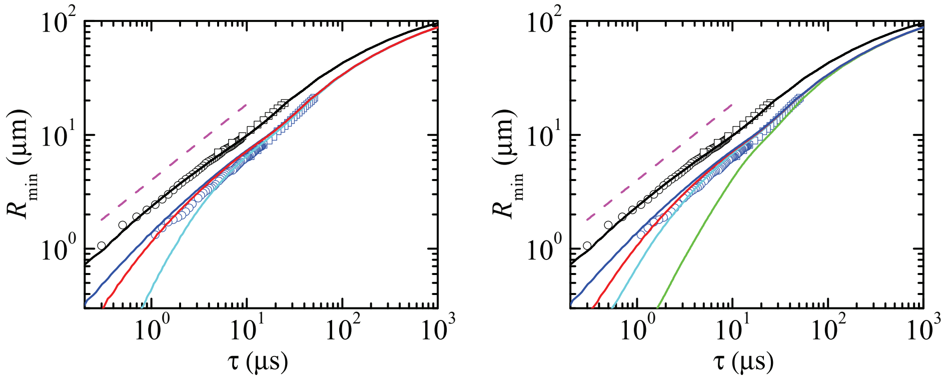

Figure 1. $R_{\min}(\tau)$ for the breakup of a pendant drop of DIW and DIW+SDS 2cmc. The black and blue symbols are the experimental data for DIW and DIW+SDS 2cmc, respectively. The different symbols correspond to experiments visualized with different magnifications. The black solid line and magenta dashed line correspond to the simulation and the power law $R_{\min}(\tau) \sim \tau^{2/3}$ for DIW, respectively. (Left) The colored solid lines correspond to simulations of DIW+SDS 2cmc for $\mu_2^{S*} = 0$ and $\mu_1^{S*} = 0$ (blue), 5×10^{-10} (red), and 3.5×10^{-9} Pa s m (cyan). (Right) The colored solid lines correspond to simulations of DIW+SDS 2cmc for $\mu_1^{S*} = 0$ and $\mu_2^{S*} = 0$ (blue), 3.5×10^{-9} (red), 10^{-8} (cyan), and 10^{-7} Pa s m (green). All the numerical results were calculated for $B = 3.454 \times 10^{-3}$, $\text{Oh} = 0.01510$, $\widehat{\Gamma}_{\text{cmc}} = 1.000$, and $\text{Pe}^S = 7.730 \times 10^4$ (see *Methods*). In the left-hand graph, the colored solid lines correspond to $\text{Oh}_2^{S*} = 0$ and $\text{Oh}_1^{S*} = 0$ (blue), 6.618×10^{-5} (red) and, 4.633×10^{-4} (cyan). In the right-hand graph, the colored solid lines correspond to $\text{Oh}_1^{S*} = 0$ and $\text{Oh}_2^{S*} = 0$ (blue), 4.633×10^{-4} (red), 1.324×10^{-3} (cyan), and 1.324×10^{-2} (green).

Figure 3 shows the numerical results for DIW+SDS 0.8cmc with $\mu_2^{S*} = 0$ and $\mu_1^{S*} = 5 \times 10^{-10}$ (Fig. 3-left in the main text) when the total number of grid points is doubled. As can be seen, the results are practically the same in the time interval analyzed in our study.

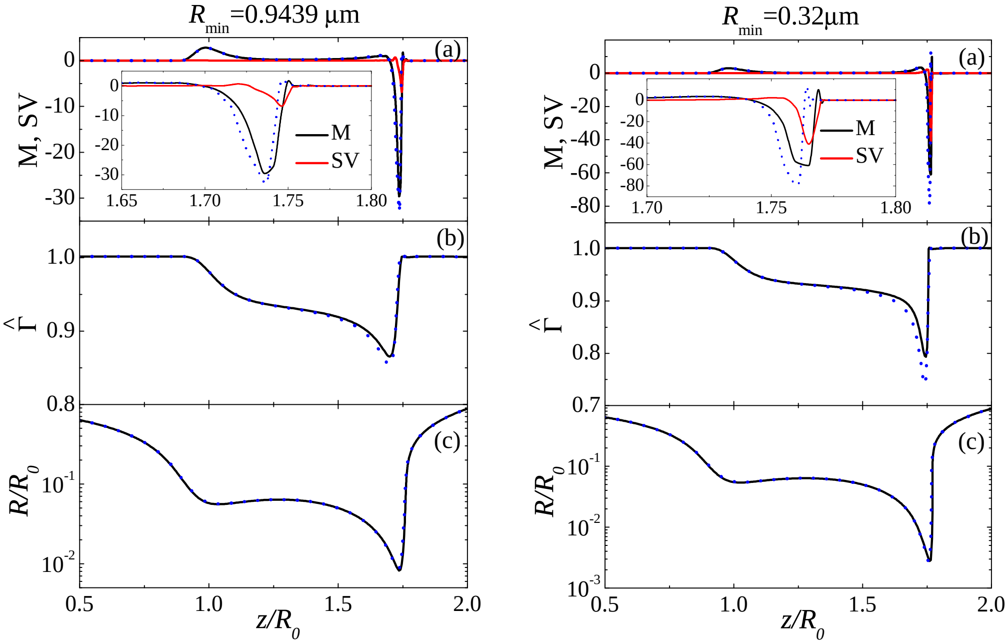

Figure 2. Axial distribution of the Marangoni stress (M) and tangential shear viscous stress (SV) (a), surfactant surface concentration (b), and free surface radius (c) for DIW+SDS 0.8cmc. The solid lines are the results for $\{\mu_1^{S*} = 5\times10^{-10}$ Pa s m, $\mu_2^{S*} = 0\}$, while the dotted lines correspond to $\mu_1^{S*} = \mu_2^{S*} = 0$. The dotted lines show the results for $\mu_1^{S*} = \mu_2^{S*} = 0$ (in the left-hand graphs, $R_{\min} = 0.9836\ \mu$m for $\mu_1^{S*} = \mu_2^{S*} = 0$). The results were calculated for $B = 3.396\times10^{-3}$, $\mathrm{Oh} = 0.01510$, $\widehat{\Gamma}_{\mathrm{cmc}} = 1.002$, $\mathrm{Pe}^S = 7.730\times10^4$, $\mathrm{Oh}_2^{S*} = 0$, and $\mathrm{Oh}_1^{S*} = 6.563\times10^{-5}$ (solid lines) and 0 (dotted lines) (see *Methods*).

Figure 4 shows the numerical results for DIW+SDS 0.8cmc with $\mu_2^{S*} = 0$ and $\mu_1^{S*} = 5\times10^{-10}$ (Fig. 3-right in the main text) when N_b is doubled. As can be seen, the results are practically the same in the time interval analyzed in our study.

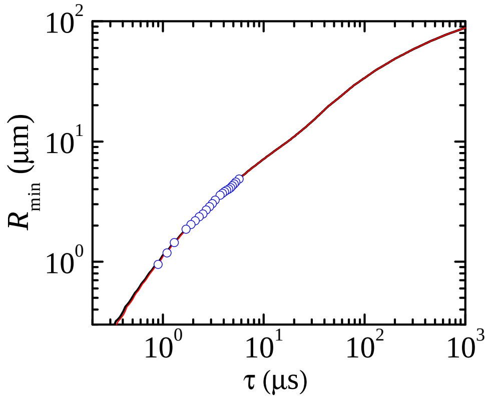

Figure 3. $R_{\mathrm{min}}(\tau)$ for the breakup of a pendant drop of DIW and DIW+SDS 0.8cmc. The blue symbols are the experimental data for DIW+SDS 0.8cmc. The red line is the simulation result for $\mu_1^{S*} = 0$ and $\mu_2^{S*} = 3.5 \times 10^{-9}$ shown in the main text. The black line corresponds to the simulation result when the total number of grid points is doubled.

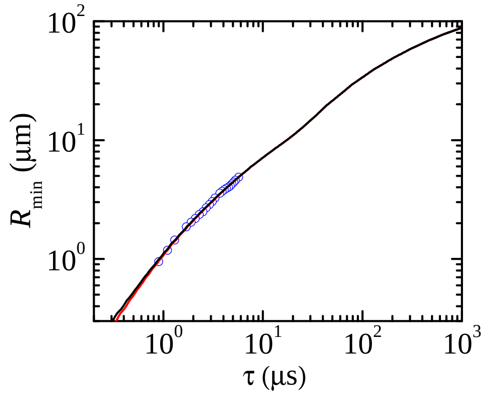

Figure 4. $R_{\mathrm{min}}(\tau)$ for the breakup of a pendant drop of DIW and DIW+SDS 0.8cmc. The blue symbols are the experimental data for DIW+SDS 0.8cmc. The red line is the simulation result for $\mu_2^{S*} = 0$ and $\mu_1^{S*} = 5 \times 10^{-10}$ shown in the main text. The black line corresponds to the simulation result when N_b is doubled.
